# Association Between Depression and Physical Conditions Requiring Hospitalization

**DOI:** 10.1001/jamapsychiatry.2023.0777

**Published:** 2023-05-03

**Authors:** Philipp Frank, G. David Batty, Jaana Pentti, Markus Jokela, Lydia Poole, Jenni Ervasti, Jussi Vahtera, Glyn Lewis, Andrew Steptoe, Mika Kivimäki

**Affiliations:** 1 Research Department of Epidemiology and Public Health, University College London, London, United Kingdom; 2 Department of Public Health, University of Turku, Turku, Finland; 3 Clinicum, Faculty of Medicine, University of Helsinki, Helsinki, Finland; 4 Finnish Institute of Occupational Health, Helsinki, Finland; 5 Department of Psychology and Logopedics, Faculty of Medicine, University of Helsinki, Helsinki, Finland; 6 School of Psychology, University of Surrey, Guildford, Surrey, United Kingdom; 7 UCL Brain Sciences, University College London, London, United Kingdom; 8 Research Department of Behavioural Science and Health, University College London, London, United Kingdom

## Abstract

**Importance:**

Depression is associated with an increased risk of physical illness, but the most common causes of hospitalization among people with depression are unclear.

**Objective:**

To examine the association of depression with an array of physical conditions requiring hospital treatment.

**Design, Setting, and Participants:**

In this outcomewide prospective multicohort study, primary analysis was based on data from the UK Biobank, a population-based study in the United Kingdom. Analyses were repeated in an independent data set of 2 cohorts in Finland, a population-based study and an occupational cohort. Data analysis was conducted between April and September 2022.

**Exposures:**

Self-reported depression, recurrent severe major depression, recurrent moderate major depression, and a single major depressive episode.

**Main Outcomes and Measures:**

A total of 77 common health conditions ascertained from linkage data to national hospital and mortality registries.

**Results:**

The analytical sample of UK Biobank participants consisted of 130 652 individuals (71 565 women [54.8%]; 59 087 men [45.2%]; mean [SD] age at baseline, 63.3 [7.8] years). The pooled data from the Finnish replication cohorts included 109 781 participants (82 921 women [78.6%]; 26 860 men [21.4%]; mean [SD] age, 42 [10.8] years). In the main analysis, severe/moderately severe depression was associated with the incidence of 29 nonoverlapping conditions requiring hospital treatment during a 5-year follow-up. Twenty-five of these associations remained after adjustment for confounders and multiple testing (adjusted hazard ratio [HR] range, 1.52-23.03) and were confirmed in the analysis of the Finnish cohorts. These included sleep disorders (HR, 5.97; 95% CI, 3.27-10.89), diabetes (HR, 5.15; 95% CI, 2.52-10.50), ischemic heart disease (HR, 1.76; 95% CI, 1.36-2.29), chronic obstructive bronchitis (HR, 4.11; 95% CI, 2.56-6.60), bacterial infections (HR, 2.52; 95% CI, 1.99-3.19), back pain (HR, 3.99; 95% CI, 2.96-5.38), and osteoarthritis (HR, 1.80; 95% CI, 1.46-2.20). The highest cumulative incidence was observed for endocrine and related internal organ diseases (245 per 1000 persons with depression; risk difference relative to unaffected individuals: 9.8%), musculoskeletal diseases (91 per 1000 persons; risk difference, 3.7%), and diseases of the circulatory system and blood (86 per 1000 persons; risk difference, 3.9%). The cumulative incidence was lower for hospital-treated mental, behavioral, and neurological disorders (20 in 1000 persons; risk difference, 1.7%). Depression was also associated with disease progression in people with prevalent heart disease or diabetes, and for 12 conditions, there was evidence of a bidirectional relationship.

**Conclusions and Relevance:**

In this study, the most common causes of hospitalization in people with depression were endocrine, musculoskeletal, and vascular diseases, not psychiatric disorders. These findings suggest that depression should be considered as a target for the prevention of physical and mental disease.

## Key Points

**Question:**

What are the most common conditions requiring hospital treatment in people with depression?

**Findings:**

In this cohort study that included 240 433 individuals, depression was associated with an increased risk of 29 hospital-treated conditions. For 12 of these conditions, there was evidence for a bidirectional relationship with depression; the highest cumulative incidence was observed for diseases of the endocrine, musculoskeletal, and circulatory systems.

**Meaning:**

In this study, the main causes of excess hospitalizations in people with depression were endocrine, musculoskeletal, and vascular diseases.

## Teaser

This cohort study examines data from the UK Biobank and 2 Finnish cohorts to gauge the association between depression and an array of physical conditions requiring hospital treatment.

## Introduction

Mental health problems are a major contributor to disease burden in high-income countries, with depression ranking among the top 10 causes of years lost to disability.^1^ While major depressive disorder (MDD) affects approximately 5% of the general population,^2^ its prevalence has been found to be markedly higher in patients with chronic medical conditions, such as myocardial infarction (29%),^3,4^ type 2 diabetes (28%),^5^ Parkinson disease (23%),^6^ stroke (18%),^7,8^ cancer (16%),^9,10^ and Alzheimer disease (13%).^11^ There is growing evidence that depression may act as an etiological risk factor for the development of specific physical illnesses^12,13,14,15,16,17,18^ and that comorbid depression may exacerbate disease progression.^19,20,21^ It has also been hypothesized that some depression-disease associations may be bidirectional, including those of depression with coronary heart disease,^18,22^ stroke,^16,23^ and diabetes.^15,24^

To date, the associations of depression with a full range of serious diseases are not well understood. Existing studies on multiple disease outcomes are often characterized by smaller sample sizes,^25,26,27^ thereby limiting the array of end points under study. Furthermore, these investigations did not assess the role of depression severity in disease risk, evaluate the extent to which the associations between depression and diseases are bidirectional, or examine the most common causes of hospitalizations in people with depression.^14,25,26,27,28,29^

Using data from the UK Biobank, a prospective cohort study, we conducted a large-scale, outcomewide investigation of the association of self-reported and physician-diagnosed depression measures and subtypes with the incidence of 77 common health conditions requiring hospital treatment. To test reproducibility of our findings, we repeated analyses in an independent study population drawn from a country with a different health care system.

## Methods

### Study Population

**Figure 1.**
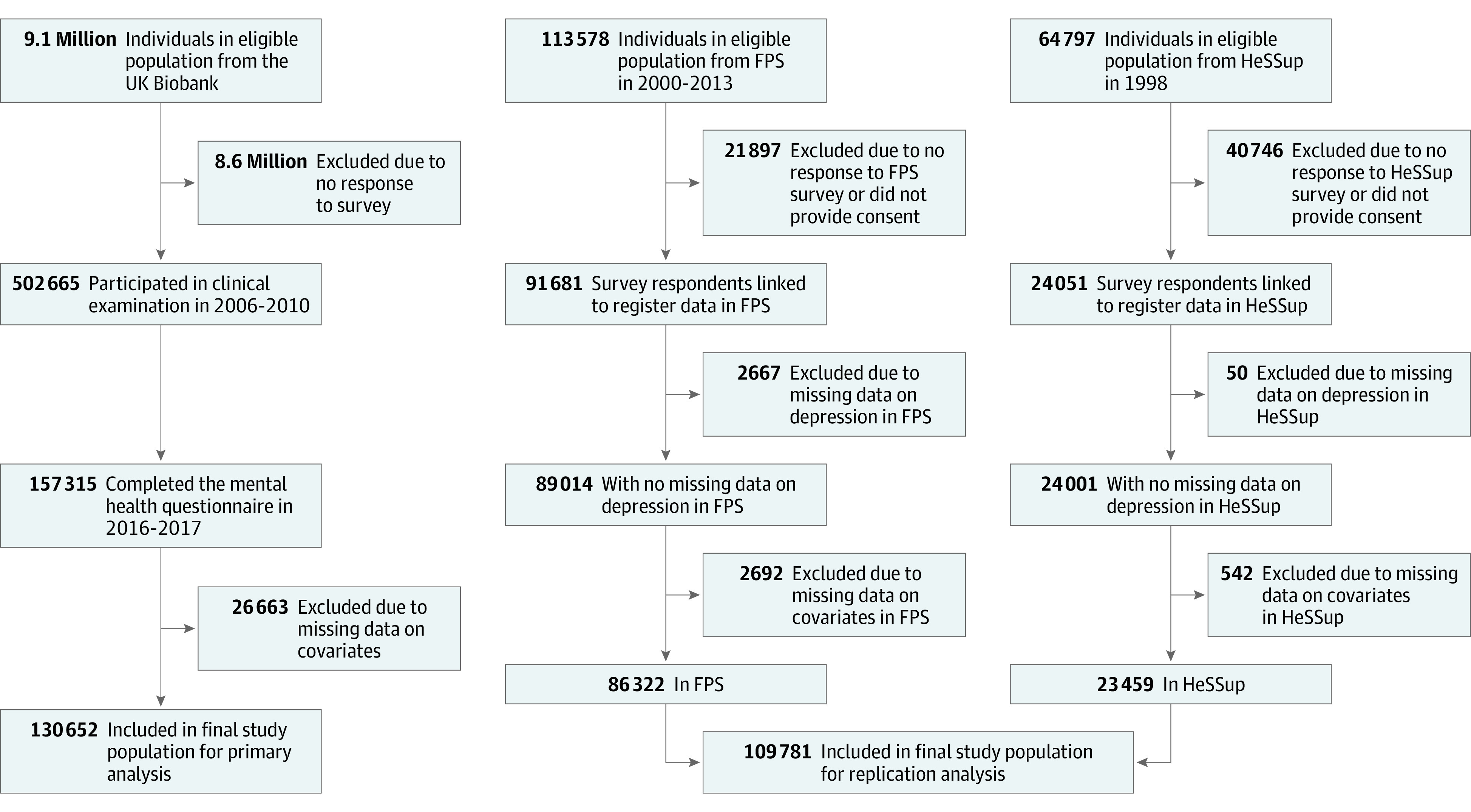
Study Profile FPS indicates Finnish Public Sector Study; HeSSup, Health and Social Support Study.

UK Biobank^30^ is a large prospective cohort study that identified participants via the UK National Health Service (NHS) records. Of the 9.1 million adults eligible for inclusion in UK Biobank, 502 665 (273 450 women) aged 38 to 73 years participated in a baseline clinical examination between 2006 and 2010. Of these, 157 315 completed an online follow-up questionnaire on mental health in 2016-2017, and a total of 130 652 individuals (age range, 46-80 years; 71 597 women) had no missing data on covariates (Figure 1).

Analyses were repeated in a pooled data set of 2 Finnish cohort studies: the population-based Health and Social Support Study (HeSSup)^31^ and the Finnish Public Sector Study (FPS), a large-scale occupational cohort of Finnish employees.^32^ The population of HeSSup comprised 23 459 individuals (13 823 women) aged 20 to 54 years who completed a survey in 1998 through 2000. The population in FPS included 86 322 employed adults (69 098 women) aged 17 to 77 years who had completed a survey during 2000 to 2002, 2004 to 2005, 2008 to 2009, and/or 2011 to 2013.

Data collection in UK Biobank was approved by the NHS National Research Ethics Service, in HeSSup by the Turku University Central Hospital Ethics Committee, and in FPS by the ethics committee of the Finnish Institute of Occupational Health. In all 3 studies, written informed consent was given prior to participation in data collection and additionally provided for register linkage. This study followed the Enhancing the Quality and Transparency of Health Research (EQUATOR) reporting guidelines.

### Assessment of Depression and Covariates at Baseline

In UK Biobank, depression was ascertained from the 9-item version of the Patient Health Questionnaire (PHQ-9), which assesses how often over the past 2 weeks an individual experienced each of the 9 depressive symptoms embodied in the *DSM-IV* (0 for not at all, 1 for several days, 2 for more than half the days, and 3 for nearly every day).^33^ Total scores (range, 0-27) were computed by summing individual item responses, and thereafter, 3 severity categories were derived: no depression (0-4, reference group), mild to moderate depression (5-14), and severe or moderately severe depression (≥15). In addition, we computed a binary depression variable (yes, no) with scores of 10 or higher indicating depression.^34^

Further information on depression was obtained from 3 previously established, UK Biobank–based depression indicators,^35^ denoting probable recurrent severe major depression (yes, no), probable recurrent moderate major depression (yes, no), and probable single major depression episode (yes, no). (Details of the methods used to derive and operationalize these variables have been described by the UK Biobank.^36^) Data on these measures were collected from participants’ responses to a touchscreen questionnaire at recruitment to UK Biobank. Questions were based on the Structured Clinical Interview for *DSM-IV* Axis I Disorders. While the focus of this study was on unipolar depression measures, we also report data on probable bipolar depression (yes, no); these results are shown in eTables 5, 8, and 15 in Supplement 1.

In HeSSup and FPS, available measures of depression included self-reported physician-diagnosed depression (yes, no) and a history of recorded hospitalizations with a depression diagnosis (yes, no, based on codes F32-F33 from the *International Statistical Classification of Diseases and Related Health Problems, Tenth Revision* [*ICD-10*]) drawn from national hospital admission registers.

We used several covariates captured at baseline. Sociodemographic variables were age and sex. Educational qualification (low, medium, high) was used as an indicator of socioeconomic position. Health behaviors included self-reported smoking status (never, previous, current), alcohol consumption (none/low, medium, high), and physical activity (physically active, not active). In UK Biobank, self-reported ethnic origin (White or non-White) was added as an additional covariate. Participants chose among the categories Asian/Asian British, Black/Black British, Chinese, White, mixed (multiracial), and other ethnic group. With most UK Biobank participants having White ethnic origin, there were too few health events at follow-up to facilitate analyses across individual minority groups. We therefore categorized these data as White or non-White.

### Ascertainment of Incident Hospital-Treated Conditions at Follow-up

In UK Biobank, newly developed physical and mental health conditions requiring hospital treatment were ascertained from linkage data to the UK National Health Service (NHS) Hospital Episode Statistics database for hospital admissions and the NHS Central Registry for mortality, from March 1995 until May 2021. In HeSSup and FPS, participants were linked to national hospital discharge and mortality registries. These electronic health records provided information on the cause and date of hospital discharge or mortality, or both, from January 1996 to December 2018. The mean (SD) follow-up was 4.6 (0.14) years in UK Biobank, 12.5 (3.93) years in FPS, and 13.8 (1.26) years in HeSSup.

In all 3 cohort studies, individual diseases and disease categories were coded according to the *ICD-10*. We investigated 77 predefined *ICD-10* disease chapters and diagnostic groups designed for outcomewide analyses.^37,38^ For each health outcome, participants with the disease at or before baseline, as ascertained from the linked health registry data, were excluded from the analysis of incident hospital-treated conditions.

### Statistical Analysis

In primary analyses of UK Biobank participants, and after confirmation that the proportional hazards assumption had not been violated (eTables 1, 2, 3, and 4 in Supplement 1), we performed separate Cox proportional hazards regression models^39^ to examine the associations of each depression measure with 77 incident disease outcomes. Hazard ratios (HRs) and accompanying 95% CIs were adjusted for age and sex and, additionally, ethnic origin, education, smoking, alcohol consumption, and physical activity. To identify the most robust associations,^40^ subsequent analyses were restricted to associations that remained statistically significant at a Bonferroni-corrected α level of *P* < 6.49 × 10^−4^ (ie, adjustment for 77 tests) and had an HR greater than or equal to 1.50. In a sensitivity analysis, we accounted for missing data on covariates by producing a multiple imputation model based on chained equations (n = 20).^41^ To examine total disease burden over time in individuals with severe/moderately severe depression, we calculated the cumulative incidence per 1000 persons for disease categories that were most consistently associated with depression.

To investigate whether depression was associated with disease progression, we examined the risk of being hospitalized because of circulatory conditions in a subgroup of UK Biobank participants with self-reported heart problems but no record of hospitalization due to circulatory conditions at baseline (n = 23 509) and the risk of being hospitalized because of diabetes in a subgroup of UK Biobank participants with diabetes who had no record of hospitalizations due to this condition at baseline (n = 4161).

To examine the reproducibility of the associations between depression and incident disease, we repeated analyses in pooled data from HeSSup and FPS by computing HRs and 95% CIs with the same statistical adjustments and for the same 77 disease end points. In these analyses, cohort was used as an additional covariate. To examine the bidirectionality of robust depression-disease associations in a subpopulation of 57 166 FPS participants with repeat data on both depression and diseases, we used multivariable-adjusted logistic regression analyses and explored whether participants with the hospital-treated disease of interest but no depression at baseline were at increased risk of developing depression at follow-up relative to individuals without depression and the disease at baseline.

All UK Biobank analyses were conducted using Stata version 17.0 and those of FPS and HeSSup using SAS version 9.4. Statistical code is provided in the eMethods in Supplement 1.

## Results

The analytical sample of UK Biobank participants consisted of 130 652 individuals (71 565 women [54.8%]; 59 087 men [45.2%]) with a mean (SD) age of 63.3 (7.8) years. A total of 104 243 participants (79.8%) had no depression, 23 843 (18.2%) reported mild to moderate depression, and 2566 (2.0%) severe or moderately severe depression (Table 1). Differences in characteristics between included and excluded participants are shown in eTable 6 in Supplement 1.

**Table 1.** Baseline Characteristics of the Primary and Replication Cohorts

	No. (%)	
	UK Biobank (primary analysis)^a^	Finnish cohorts (replication analysis)
No. of participants	130 652	109 781
Age, mean (SD), y	63.3 (7.8)	42.0 (10.8)
Sex		
Men	59 087 (45.2)	26 860 (21.4)
Women	71 565 (54.8)	82 921 (78.6)
Depression		
None	104 243 (79.8)	NA
Mild/moderate	23 843 (18.2)	NA
Severe/moderately severe	2566 (2.0)	NA
Self-reported physician-diagnosed depression		
No	NA	96 655 (88.0)
Yes	NA	13 126 (12.0)
Hospitalization due to depression		
No	NA	109 289 (99.5)
Yes	NA	492 (0.5)
Ethnic origin^b^		
White	126 938 (97.2)	NA
Non-White	3714 (2.8)	NA
Education		
None/elementary	7996 (6.1)	14 964 (13.6)
Secondary	60 662 (46.4)	41 379 (37.7)
Tertiary	61 994 (47.5)	53 438 (48.7)
Smoking		
Never	75 080 (57.5)	65 469 (59.6)
Previous	46 196 (35.3)	23 150 (21.1)
Current	9376 (7.2)	21 162 (19.3)
Alcohol use		
None	18 321 (14.0)	16 555 (15.1)
Low	46 407 (35.5)	82 282 (75.0)
Moderate	34 739 (26.6)	4584 (4.2)
High	31 185 (23.9)	6360 (5.8)
Physically inactive		
Yes	69 871 (53.5)	21 982 (20.0)
No	60 781 (46.5)	87 799 (80.0)

Abbreviation: NA, not applicable.

^a^ The sample with scores from the 9-item Patient Health Questionnaire from UK Biobank.

^b^ Ethnic origin was self-reported by UK Biobank participants and then categorized by researchers from the choices Asian/Asian British, Black/Black British, Chinese, White, mixed (multiracial), and other ethnic group.

**Figure 2.**
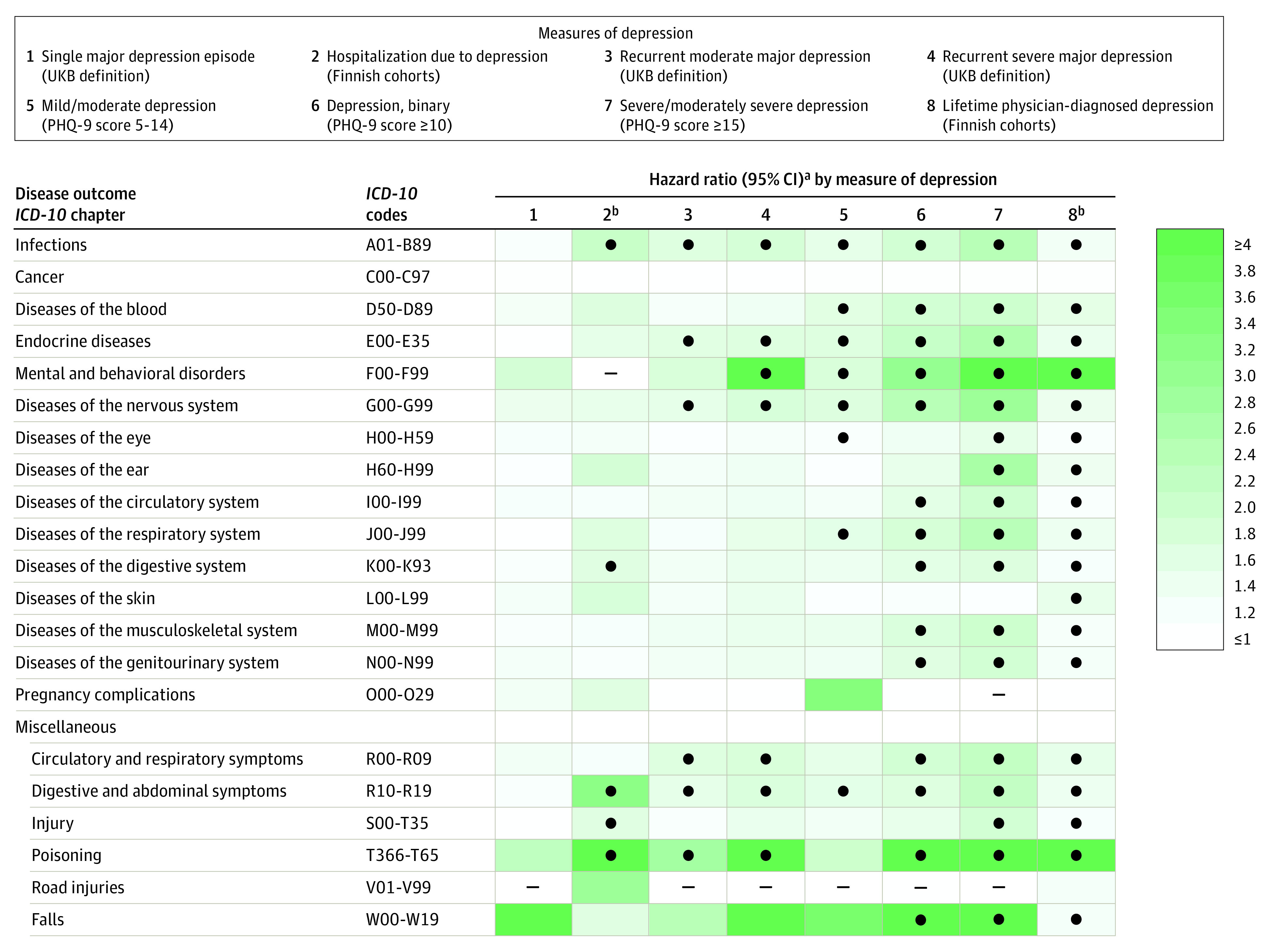
Associations Between Measures of Depression and Main *ICD-10* Disease Groups in Individuals From the UK Biobank (UKB) and 2 Finnish Cohorts, the Health and Social Support Study (HeSSup) and Finnish Public Sector Study (FPS) The ● in the table indicates significance at *P* < .001 (Bonferroni correction for multiple testing); the – indicates analyses were not possible for this condition because of the low number of cases. *ICD-10* indicates *International Statistical Classification of Diseases and Related Health Problems, Tenth Revision*; PHQ-9, 9-item Patient Health Questionnaire. ^a^Hazard ratio for depression as predictor of disease adjusted for age, sex, education, self-reported ethnic origin, smoking, alcohol, and physical activity at baseline. ^b^Analysis in Finnish cohorts. Analyses on other measures of depression were based on UK Biobank data.

In Figure 2, we summarize the multivariable-adjusted associations of each depression measure with main *ICD-10* disease groups according to strength and statistical significance in UK Biobank (results for all 77 disease outcomes are reported in eTables 7-11 and 16 in Supplement 1). After adjustment for age, sex, ethnic origin, education, smoking status, alcohol consumption, and physical activity, severe/moderately severe depression according to PHQ-9 score was most strongly associated with incident disease. The effect estimates for mild to moderate depression (PHQ-9) and a probable single major depression episode (UK Biobank definition) were smaller. In analyses using an alternative PHQ-9 cutoff of 10 or higher, the effect sizes were between those of mild/moderate depression and severe/moderately severe depression.

In Table 2, we show the associations of severe/moderately severe depression with 29 nonoverlapping disease outcomes that were robust to multivariable adjustment, had an HR of greater than or equal to 1.50, and were statistically significant at a Bonferroni corrected α level, *P* < 6.49 × 10^−4^ in UK Biobank. Individuals with severe/moderately severe depression experienced an increased risk of hospitalizations due to poisoning, falls, and injuries (range of HRs, 1.86-23.03); diseases of the endocrine, genitourinary, and digestive systems (HRs, 1.67-6.97); and mental and behavioral disorders and diseases of the nervous system (HRs, 3.67-16.47). They also experienced a more than 1.5-times increased risk for diseases of the musculoskeletal system (HRs, 1.80-6.80); diseases of the respiratory system (HRs, 2.65-4.11); diseases of the circulatory system, blood, and related symptoms (HRs, 1.76-4.38); diseases of the ear or eye (HRs, 1.52-2.76); and infections and diseases of the skin (HRs, 2.01-2.52). Use of multiple imputation analysis to supplement missing values on covariates did not materially change the results (eResults 1 and eTable 12 in Supplement 1).

**Table 2.** Bidirectional Associations Between Depression and Hospital-Treated Health Conditions (UK Biobank and Finnish Cohorts)

Diagnosis or diagnostic group by category^a^	Depression at baseline → disease at follow-up, HR (95% CI)^b^		Disease at baseline → depression at follow-up, self-reported physician-diagnosed depression, OR (95% CI)^b^
	Severe or moderately severe depression	Self-reported physician-diagnosed depression	
Poisoning, falls, and injuries			
Poisoning^c^	8.63 (5.04-14.76)	5.22 (4.38-6.22)	3.20 (1.78-5.81)
Falls^c^	23.03 (4.58-115.85)	1.23 (1.13-1.34)	1.21 (1.01-1.45)
Injuries	1.86 (1.54-2.26)	1.20 (1.13-1.27)	1.08 (0.94-1.23)
Diseases of the endocrine, genitourinary, and digestive systems and symptoms			
Obesity requiring hospital treatment	6.97 (2.75-17.65)	2.45 (1.95-3.08)	2.33 (0.76-7.13)
Diabetes	5.15 (2.52-10.50)	1.38 (1.29-1.47)	1.11 (0.86-1.44)
Kidney failure	3.66 (2.30-5.83)	1.24 (0.92-1.66)	0.98 (0.30-3.22)
Digestive and abdominal symptoms^c^	2.15 (1.80-2.57)	1.37 (1.23-1.53)	1.54 (1.27-1.86)
Diseases of the digestive system^c^	1.67 (1.49-1.87)	1.20 (1.14-1.27)	1.20 (1.07-1.35)
Mental and behavioral disorders, diseases of the nervous system			
Mood disorders^c^	5.29 (2.17-12.86)	5.85 (5.10-6.71)	5.38 (2.98-9.70)
Neurotic, stress-related, and somatoform disorders	5.10 (2.26-11.53)	6.78 (6.04-7.61)	1.45 (0.65-3.22)
Sleep disorders^c^	5.97 (3.27-10.89)	2.07 (1.88-2.27)	2.10 (1.48-2.97)
Headaches	3.67 (2.16-6.25)	1.82 (1.44-2.31)	1.42 (0.77-2.61)
Parkinson disease	16.47 (6.77-40.06)	1.29 (0.93-1.79)	1.22 (0.28-5.32)
Diseases of the musculoskeletal system			
Back pain	3.99 (2.96-5.38)	1.70 (1.42-2.03)	1.16 (0.74-1.80)
Gout	6.80 (2.32-19.93)	1.22 (0.86-1.73)	1.56 (0.66-3.68)
Sciatica^c^	2.73 (1.78-4.19)	1.19 (1.03-1.37)	1.77 (1.40-2.24)
Rheumatoid arthritis and related disorders	2.54 (1.50-4.30)	1.05 (0.93-1.18)	0.73 (0.56-0.95)
Osteoarthritis	1.80 (1.46-2.20)	1.21 (1.13-1.30)	1.15 (0.91-1.46)
Soft tissue disorders^c^	1.81 (1.41-2.32)	1.18 (1.09-1.27)	1.47 (1.25-1.73)
Diseases of the respiratory system			
Chronic obstructive bronchitis	4.11 (2.56-6.60)	1.69 (1.37-2.07)	2.18 (0.73-6.53)
Influenza and pneumonia	2.65 (2.01-3.50)	1.48 (1.33-1.64)	1.07 (0.76-1.50)
Diseases of the circulatory system and blood and related symptoms			
Heart failure	4.38 (2.66-7.23)	1.30 (1.05-1.62)	1.59 (0.67-3.78)
Circulatory and respiratory symptoms^c^	2.16 (1.78-2.62)	1.49 (1.33-1.67)	1.39 (1.07-1.80)
Anemia	2.01 (1.50-2.70)	1.40 (1.09-1.81)	1.28 (0.61-2.69)
Ischemic heart diseases	1.76 (1.36-2.29)	1.24 (1.12-1.37)	1.23 (0.89-1.72)
Diseases of the ear or eye			
Diseases of the ear	2.67 (1.70-4.18)	1.37 (1.17-1.61)	0.96 (0.67-1.37)
Diseases of the eye	1.52 (1.28-1.81)	1.15 (1.07-1.24)	0.95 (0.71-1.28)
Infections and diseases of the skin			
Skin infections and eczema	2.01 (1.36-2.97)	1.39 (1.13-1.72)	1.25 (0.82-1.92)
Bacterial infections^c^	2.52 (1.99-3.19)	1.32 (1.20-1.46)	1.51 (1.18-1.94)

Abbreviations: FPS, Finnish Public Sector Study; HR, hazard ratio; OR, odds ratio; PHQ-9, 9-item Patient Health Questionnaire.

^a^ Ordered by strength of association between depression at baseline and incident disease at follow-up (average rank order).

^b^ Hazard ratios, ORs, and 95% CIs are adjusted for age, sex, education, self-reported ethnic origin (UK Biobank), smoking, alcohol, and physical activity at baseline. Data on severe or moderately severe depression (PHQ-9) are from UK Biobank (comparator group is individuals without depression) and those for self-reported physician-diagnosed depression from the Finnish cohorts.

^c^ Bidirectional association confirmed in FPS.

In analyses exploring whether depression was associated with disease progression in UK Biobank participants with prevalent heart conditions, individuals with mild/moderate depression had a 1.26-times increased risk (95% CI, 1.14-1.40) and those with severe/moderately severe depression a 1.92-times increased risk (95% CI, 1.52-2.43) of hospitalizations due to circulatory conditions at follow-up relative to participants without depression at baseline. Similar results were found in a subgroup of participants with self-reported physician-diagnosed diabetes at baseline who had not been hospitalized because of the condition. Relative to participants without depression at baseline, individuals with mild/moderate depression had a 2.35-times increased risk (95% CI, 1.38-3.98), and those with severe/moderately severe depression a 3.60-times higher risk (95% CI, 1.46-8.90) of hospitalization due to diabetes at follow-up (eResults 2 in Supplement 1).

**Figure 3.**
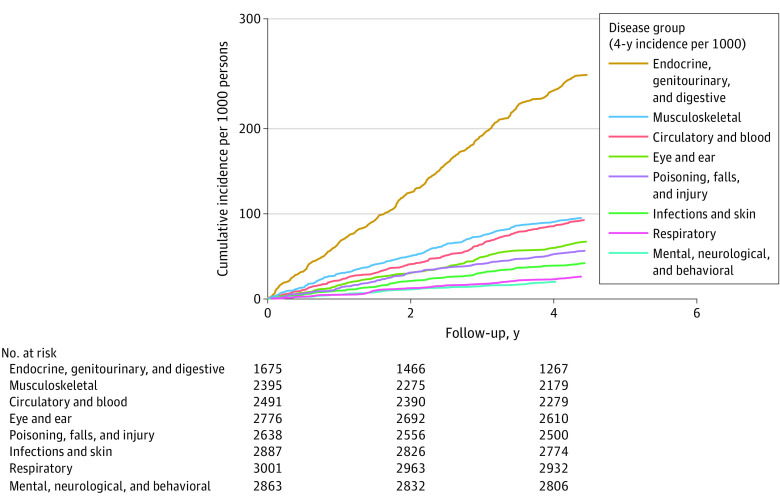
Four-Year Cumulative Incidence for 8 Disease Categories Among People With Severe/Moderately Severe Depression (UK Biobank Cohort)

To describe the burden of depression-related comorbidities over time in absolute terms in UK Biobank, we computed the cumulative incidence during the follow-up for 8 disease categories in people with severe/moderately severe depression (Figure 3). These categories combine conditions that were most consistently associated with this depression measure after adjustment for covariates and multiple testing (Table 2). The highest 4-year cumulative incidence and the greatest difference in cumulative incidence between participants with and without severe/moderately severe depression were observed for endocrine and related internal organ diseases, with 245 per 1000 persons with depression vs 147 per 1000 persons without depression at baseline requiring hospital treatment because of these diseases (absolute excess risk compared with people without depression, 9.8%). Musculoskeletal diseases ranked second (4-year incidence, 91 per 1000 persons with depression; absolute excess risk, 3.7%), and diseases of the circulatory system and blood third (86 per 1000 persons; absolute excess risk, 3.9%). Mental, behavioral, and neurological disorders had a lower 4-year cumulative incidence, with only 20 in 1000 persons with depression requiring hospital treatments because of mood disorders; neurotic, stress-related, and somatoform disorders; sleep disorders; headaches; or Parkinson disease during the 4-year follow-up (absolute excess risk, 1.7%). The absolute risk difference for people with vs those without severe/moderately severe depression across all 29 conditions was 16.3 percentage points (eTable 14 in Supplement 1).

The pooled data from the replication cohorts, HeSSup and FPS, included 109 781 participants (82 921 women [78.6%]; 26 860 men [21.4%]) with a mean (SD) age of 42 (10.8) years. Of these, 13 126 participants (12.0%) reported physician-diagnosed depression, and 492 (0.5%) had been hospitalized because of a depression diagnosis at baseline (Table 1). Physician-diagnosed depression in the replication cohorts was almost as strongly associated with incident disease as severe/moderately severe depression in UK Biobank. Accordingly, robust associations of physician-diagnosed depression were confirmed for 25 of the 29 nonoverlapping diseases that were associated with severe/moderately severe depression in UK Biobank participants (Figure 2 and Table 2). The patterns of associations were similar for people who were hospitalized because of depression, albeit with smaller effect estimates (Figure 2).

In an analysis of repeat data from 57 166 FPS participants (Table 2, right column, and eTable 13 in Supplement 1), we found support for a bidirectional association of depression with poisonings, falls, digestive and abdominal symptoms, diseases of the digestive system, mood disorders, sleep disorders, sciatica, soft tissue disorders, circulatory and respiratory symptoms, and bacterial infections, with odds ratios ranging from 1.2 (diseases of the digestive system) to 5.4 (mood disorders).

## Discussion

The findings of this multicohort study suggest that, compared with individuals without depression, those with self-reported severe or moderately severe depression have at least a 1.5-times higher risk of 29 nonoverlapping conditions across multiple organ systems. The greatest absolute risk was observed for endocrine and related internal organ diseases, followed by musculoskeletal diseases and diseases of the circulatory system and blood. Among people with depression, the cumulative incidence for hospital-treated mental disorders was lower, possibly due to conditions such as mood and anxiety disorders being predominantly treated in primary care. There was also evidence for bidirectional depression-disease relationships, such that poisonings; falls; diseases or symptoms of the circulatory, respiratory, digestive, and musculoskeletal systems; and severe infections were associated with subsequent onset of depression.

By applying an outcomewide approach to a single analytical setting, this study provides novel insights into depression-related hospitalizations and also confirms the results of several previous single-outcome studies. Overall, our findings emphasize the important role of depression as a risk factor for physical illnesses requiring hospital treatment. The observed elevated risk for poisonings; headaches; neurotic, stress-related, and somatoform disorders; sleep disorders; obesity; back pain; and chronic obstructive bronchitis in individuals with depression confirm previous meta-analytic investigations and large-scale studies.^27,28,29,42,43^ Our results also support earlier evidence on the prospective links between depression and diabetes,^15^ Parkinson disease,^12^ and heart disease.^27,44,45^ In addition, they are in agreement with a recent large-scale study of around 6 million people that captured data on mood disorders and subsequent medical diseases from linkage to national registries.^28^ In that study, in accordance with our findings, strong associations were observed with diseases of the blood and endocrine system and weak or nonexisting associations with cancers. It has been hypothesized that depression may also affect disease progression, for example, by reducing adherence to prescribed treatments or poorer self-care.^20,21,46^ We found that depression was associated with worse disease prognosis in people with self-reported heart problems and diabetes at baseline.

There are several plausible mechanisms for the observed associations. Behavioral pathways may involve smoking, particularly in relation to diseases such as chronic obstructive bronchitis^47^ and heart disease^46^; lack of physical activity for conditions such as obesity, diabetes, cardiovascular disease, and musculoskeletal disorders; and high alcohol consumption in relation to diseases of the endocrine, digestive, genitourinary, and circulatory systems.^46,48^ There is also some evidence pointing toward shared genetic variation between depression and an array of neurological, circulatory, musculoskeletal, and respiratory disorders.^29^ Further shared biological mechanisms that may underlie multiple comorbid conditions in depression include alterations in levels of monoamines, which have been linked to sleep problems^49^ and pain^50^; as well as a dysregulated hypothalamic-pituitary-adrenal axis for cardiometabolic diseases.^46,51^ Another plausible mechanism is inflammation. A recent case-control analysis of UK Biobank participants suggests that levels of systemic inflammation were significantly higher in people with depression compared with the reference group without depression.^52^ Systemic inflammation has also been found to contribute to a wide range of conditions, including obesity^53^ and cardiovascular disease.^46^ Lastly, the observed bidirectional associations in our study demonstrate that severe physical illnesses may be associated with poor mental health.

In recent years, there has been emerging interest in precision medicine approaches to psychiatry. In our study, the identified depression-disease associations were largely consistent across depression measures, although there was evidence for a dose-response effect by severity and chronicity. We also observed widespread but slightly weaker depression-disease associations for inflammation-related^54^ and obesity-related^55^ symptom profiles, as well as for probable bipolar depression, suggesting that most depression-disease associations persist across varying symptom expressions (eTable 8 in Supplement 1). Thus, our findings support only partially the notion that different symptoms may have distinct underlying etiological pathways that contribute to variability in associations with disease pathologies.^51,54^ These hypotheses warrant further testing in future large-scale studies.

### Limitations

Interpretation of our findings requires consideration of various limitations. First, causal inference is not possible given that all the studies included in the present analysis are observational. Conclusions about the directions of associations may also be hampered owing to missing data on undiagnosed diseases or disease diagnoses made in primary care. In addition, the utilization of data-driven HR thresholds (1.5) and a predefined set of common diseases with public health relevance may have resulted in an underestimation of the actual hospitalization burden associated with depression. Second, while we were able to test disease associations for various presentations of depression, no data were available on other subtypes, such as dysthymia or seasonal affective disorder. This highlights the need for future investigations to explore whether these subtypes are differentially related to disease risk.

Third, despite being directionally consistent, effect estimates for some depression-disease associations were higher in UK Biobank than in the Finnish cohorts. There are at least 2 explanations for these differences. The participants of FPS are employees, and there is evidence suggesting that occupational cohorts have more favorable risk factor profiles and a lower disease incidence compared with the general population.^56^ Another plausible explanation is that the primary exposure in UK Biobank was severe/moderately severe depression, whereas severity levels of depression were not considered in the Finnish cohorts. It may be that the Finnish depression measure also captured milder cases of depression, which may explain some of the lower effect estimates observed in the analyses of the Finnish cohorts. Fourth, with the large majority of participants in the present studies having a White ethnic background, the generalizability of our results to minority groups is unknown.

## Conclusions

Our findings show that the associations of depression with physical disease are widespread across multiple organ systems. The most common causes of hospitalization in our cohort among people with depression were endocrine, musculoskeletal, and vascular diseases. This suggests that depression should be considered more widely as a target for somatic disease prevention and treatment.

## Notes

Supplement 1. **eTable 1.** Test of the Cox proportional hazards assumptions (PHQ-9) **eTable 2.** Test of the Cox proportional hazards assumptions (UKB severe MDD) **eTable 3.** Test of the Cox proportional hazards assumptions (UKB moderate MDD) **eTable 4.** Test of the Cox proportional hazards assumptions (UKB single MDD episode) **eTable 5.** Test of the Cox proportional hazards assumptions (UKB bipolar depression) **eMethods.** Statistical Code STATA. Loop for Cox proportional hazards regression analyses (example, UK Biobank) **eTable 6.** Baseline characteristics of included versus excluded participants (primary cohort) **eTable 7.** Multivariable-adjusted associations between measures of depression and the incidence of 77 diseases (UK Biobank and Finnish cohorts) **eTable 8.** Multivariable-adjusted associations between measures of depression and the incidence of 77 diseases (UK Biobank) **eTable 9.** Multivariable-adjusted associations between measures of depression and the incidence of 77 diseases (Finnish cohorts) **eTable 10.** Multivariable-adjusted associations between depression (PHQ-9, cut-off 10 or higher to indicate depression) and incident disease (UK Biobank) **eTable 11.** Multivariable-adjusted associations between one standard deviation increment in the original PHQ-9 score and incident disease (UK Biobank) **eResults 1.** Hazard ratios for the association of severe/moderately severe depression (PHQ-9) with incident diseases (robust depression-disease associations) in UK Biobank before and after multiple imputation **eTable 12.** Multivariable-adjusted associations between severe/moderately severe depression and incident disease after multiple imputation (UK Biobank) **eResults 2.** The association of depression with disease progression in UK Biobank participants with prevalent heart problems or diabetes **eTable 13.** Multivariable-adjusted Association of 77 health conditions with depression risk (Finnish Public Sector study) **eTable 14.** 4-year cumulative incidence per 1000 persons for 8 disease categories among people with and without depression (subtypes) **eTable 15.** Hazard ratios for the association of 8 depression measures with incident disease (UK Biobank and Finnish cohorts) **eTable 16.** Frequencies and cases per depression measure **eAppendix.** STROBE Statement

Supplement 2. Data sharing statement

## References

[1] Reiner RC Jr, Olsen HE, Ikeda CT, ; GBD 2017 Child and Adolescent Health Collaborators. Diseases, injuries, and risk factors in child and adolescent health, 1990 to 2017: findings from the Global Burden of Diseases, Injuries, and Risk Factors 2017 Study. JAMA Pediatr. 2019;173(6):e190337-e190337. doi:10.1001/jamapediatrics.2019.0337 31034019 PMC6547084

[2] Vos T, Abajobir AA, Abate KH, ; GBD 2016 Disease and Injury Incidence and Prevalence Collaborators. Global, regional, and national incidence, prevalence, and years lived with disability for 328 diseases and injuries for 195 countries, 1990-2016: a systematic analysis for the Global Burden of Disease Study 2016. Lancet. 2017;390(10100):1211-1259. doi:10.1016/S0140-6736(17)32154-2 28919117 PMC5605509

[3] Doyle F, McGee H, Conroy R, . Systematic review and individual patient data meta-analysis of sex differences in depression and prognosis in persons with myocardial infarction: a MINDMAPS study. Psychosom Med. 2015;77(4):419-428. doi:10.1097/PSY.0000000000000174 25886829

[4] Feng L, Li L, Liu W, . Prevalence of depression in myocardial infarction: a PRISMA-compliant meta-analysis. Medicine (Baltimore). 2019;98(8):e14596. doi:10.1097/MD.0000000000014596 30813183 PMC6407970

[5] Khaledi M, Haghighatdoost F, Feizi A, Aminorroaya A. The prevalence of comorbid depression in patients with type 2 diabetes: an updated systematic review and meta-analysis on huge number of observational studies. Acta Diabetol. 2019;56(6):631-650. doi:10.1007/s00592-019-01295-9 30903433

[6] Goodarzi Z, Mrklas KJ, Roberts DJ, Jette N, Pringsheim T, Holroyd-Leduc J. Detecting depression in Parkinson disease: a systematic review and meta-analysis. Neurology. 2016;87(4):426-437. doi:10.1212/WNL.0000000000002898 27358339 PMC4977107

[7] Ayerbe L, Ayis S, Wolfe CD, Rudd AG. Natural history, predictors and outcomes of depression after stroke: systematic review and meta-analysis. Br J Psychiatry. 2013;202(1):14-21. doi:10.1192/bjp.bp.111.107664 23284148

[8] Mitchell AJ, Sheth B, Gill J, . Prevalence and predictors of post-stroke mood disorders: a meta-analysis and meta-regression of depression, anxiety and adjustment disorder. Gen Hosp Psychiatry. 2017;47:48-60. doi:10.1016/j.genhosppsych.2017.04.001 28807138

[9] Mitchell AJ, Chan M, Bhatti H, . Prevalence of depression, anxiety, and adjustment disorder in oncological, haematological, and palliative-care settings: a meta-analysis of 94 interview-based studies. Lancet Oncol. 2011;12(2):160-174. doi:10.1016/S1470-2045(11)70002-X 21251875

[10] Krebber AM, Buffart LM, Kleijn G, . Prevalence of depression in cancer patients: a meta-analysis of diagnostic interviews and self-report instruments. Psychooncology. 2014;23(2):121-130. doi:10.1002/pon.3409 24105788 PMC4282549

[11] Chi S, Wang C, Jiang T, Zhu X-C, Yu J-T, Tan L. The prevalence of depression in Alzheimer’s disease: a systematic review and meta-analysis. Curr Alzheimer Res. 2015;12(2):189-198. doi:10.2174/1567205012666150204124310 25654505

[12] Wang S, Mao S, Xiang D, Fang C. Association between depression and the subsequent risk of Parkinson’s disease: a meta-analysis. Prog Neuropsychopharmacol Biol Psychiatry. 2018;86:186-192. doi:10.1016/j.pnpbp.2018.05.025 29859854

[13] Gao YH, Zhao HS, Zhang FR, . The relationship between depression and asthma: a meta-analysis of prospective studies. PLoS One. 2015;10(7):e0132424. doi:10.1371/journal.pone.0132424 26197472 PMC4510436

[14] Pan A, Keum N, Okereke OI, . Bidirectional association between depression and metabolic syndrome: a systematic review and meta-analysis of epidemiological studies. Diabetes Care. 2012;35(5):1171-1180. doi:10.2337/dc11-2055 22517938 PMC3329841

[15] Mezuk B, Eaton WW, Albrecht S, Golden SH. Depression and type 2 diabetes over the lifespan: a meta-analysis. Diabetes Care. 2008;31(12):2383-2390. doi:10.2337/dc08-0985 19033418 PMC2584200

[16] Pan A, Sun Q, Okereke OI, Rexrode KM, Hu FB. Depression and risk of stroke morbidity and mortality: a meta-analysis and systematic review. JAMA. 2011;306(11):1241-1249. doi:10.1001/jama.2011.1282 21934057 PMC3242806

[17] Russ TC, Stamatakis E, Hamer M, Starr JM, Kivimäki M, Batty GD. Association between psychological distress and mortality: individual participant pooled analysis of 10 prospective cohort studies. BMJ. 2012;345:e4933. doi:10.1136/bmj.e4933 22849956 PMC3409083

[18] Nicholson A, Kuper H, Hemingway H. Depression as an aetiologic and prognostic factor in coronary heart disease: a meta-analysis of 6362 events among 146 538 participants in 54 observational studies. Eur Heart J. 2006;27(23):2763-2774. doi:10.1093/eurheartj/ehl338 17082208

[19] McKay KA, Tremlett H, Fisk JD, ; CIHR Team in the Epidemiology and Impact of Comorbidity on Multiple Sclerosis. Psychiatric comorbidity is associated with disability progression in multiple sclerosis. Neurology. 2018;90(15):e1316-e1323. doi:10.1212/WNL.0000000000005302 29523642 PMC5894930

[20] Hare DL, Toukhsati SR, Johansson P, Jaarsma T. Depression and cardiovascular disease: a clinical review. Eur Heart J. 2014;35(21):1365-1372. doi:10.1093/eurheartj/eht462 24282187

[21] Moulton CD, Pickup JC, Ismail K. The link between depression and diabetes: the search for shared mechanisms. Lancet Diabetes Endocrinol. 2015;3(6):461-471. doi:10.1016/S2213-8587(15)00134-5 25995124

[22] van Melle JP, de Jonge P, Kuyper AM, ; MIND-IT investigators. Prediction of depressive disorder following myocardial infarction data from the Myocardial Infarction and Depression-Intervention Trial (MIND-IT). Int J Cardiol. 2006;109(1):88-94. doi:10.1016/j.ijcard.2005.05.053 16002163

[23] Jørgensen TS, Wium-Andersen IK, Wium-Andersen MK, . Incidence of depression after stroke, and associated risk factors and mortality outcomes, in a large cohort of Danish patients. JAMA Psychiatry. 2016;73(10):1032-1040. doi:10.1001/jamapsychiatry.2016.1932 27603000

[24] Rotella F, Mannucci E. Diabetes mellitus as a risk factor for depression: a meta-analysis of longitudinal studies. Diabetes Res Clin Pract. 2013;99(2):98-104. doi:10.1016/j.diabres.2012.11.022 23265924

[25] Scott KM, Lim C, Al-Hamzawi A, . Association of mental disorders with subsequent chronic physical conditions: world mental health surveys from 17 countries. JAMA Psychiatry. 2016;73(2):150-158. doi:10.1001/jamapsychiatry.2015.2688 26719969 PMC5333921

[26] Han X, Hou C, Yang H, . Disease trajectories and mortality among individuals diagnosed with depression: a community-based cohort study in UK Biobank. Mol Psychiatry. 2021;26(11):6736-6746. doi:10.1038/s41380-021-01170-6 34035478 PMC8145187

[27] Patten SB, Williams JV, Lavorato DH, Modgill G, Jetté N, Eliasziw M. Major depression as a risk factor for chronic disease incidence: longitudinal analyses in a general population cohort. Gen Hosp Psychiatry. 2008;30(5):407-413. doi:10.1016/j.genhosppsych.2008.05.001 18774423

[28] Momen NC, Plana-Ripoll O, Agerbo E, . Association between mental disorders and subsequent medical conditions. N Engl J Med. 2020;382(18):1721-1731. doi:10.1056/NEJMoa1915784 32348643 PMC7261506

[29] Mulugeta A, Zhou A, King C, Hyppönen E. Association between major depressive disorder and multiple disease outcomes: a phenome-wide Mendelian randomisation study in the UK Biobank. Mol Psychiatry. 2020;25(7):1469-1476. doi:10.1038/s41380-019-0486-1 31427754

[30] Collins R, UK Biobank. UK Biobank: protocol for a large-scale prospective epidemiological resource. Amended March 21, 2007. https://www.ukbiobank.ac.uk/media/gnkeyh2q/study-rationale.pdf

[31] Korkeila K, Suominen S, Ahvenainen J, . Non-response and related factors in a nation-wide health survey. Eur J Epidemiol. 2001;17(11):991-999. doi:10.1023/A:1020016922473 12380710

[32] Kivimäki M, Lawlor DA, Davey Smith G, . Socioeconomic position, co-occurrence of behavior-related risk factors, and coronary heart disease: the Finnish Public Sector study. Am J Public Health. 2007;97(5):874-879. doi:10.2105/AJPH.2005.078691 17395837 PMC1854863

[33] Gilbody S, Richards D, Brealey S, Hewitt C. Screening for depression in medical settings with the Patient Health Questionnaire (PHQ): a diagnostic meta-analysis. J Gen Intern Med. 2007;22(11):1596-1602. doi:10.1007/s11606-007-0333-y 17874169 PMC2219806

[34] Kroenke K, Spitzer RL, Williams JB. The PHQ-9: validity of a brief depression severity measure. J Gen Intern Med. 2001;16(9):606-613. doi:10.1046/j.1525-1497.2001.016009606.x 11556941 PMC1495268

[35] Smith DJ, Nicholl BI, Cullen B, . Prevalence and characteristics of probable major depression and bipolar disorder within UK Biobank: cross-sectional study of 172,751 participants. PLoS One. 2013;8(11):e75362. doi:10.1371/journal.pone.0075362 24282498 PMC3839907

[36] UK Biobank. Field descriptions and derivation for variables related to bipolar disorder, major depression status and neuroticism score. Accessed January 1, 2022. https://biobank.ndph.ox.ac.uk/showcase/ukb/docs/MentalStatesDerivation.pdf

[37] Kivimäki M, Batty GD, Pentti J, . Modifications to residential neighbourhood characteristics and risk of 79 common health conditions: a prospective cohort study. Lancet Public Health. 2021;6(6):e396-e407. doi:10.1016/S2468-2667(21)00066-9 34051163 PMC8172714

[38] Kivimäki M, Batty GD, Pentti J, . Association between socioeconomic status and the development of mental and physical health conditions in adulthood: a multi-cohort study. Lancet Public Health. 2020;5(3):e140-e149. doi:10.1016/S2468-2667(19)30248-8 32007134

[39] Cox DR. Regression models and life-tables. J R Stat Soc Series B Stat Methodol. 1972;34(2):187-202.

[40] Ioannidis JP. Why most published research findings are false. PLoS Med. 2005;2(8):e124. doi:10.1371/journal.pmed.0020124 16060722 PMC1182327

[41] White IR, Royston P, Wood AM. Multiple imputation using chained equations: issues and guidance for practice. Stat Med. 2011;30(4):377-399. doi:10.1002/sim.4067 21225900

[42] Luppino FS, de Wit LM, Bouvy PF, . Overweight, obesity, and depression: a systematic review and meta-analysis of longitudinal studies. Arch Gen Psychiatry. 2010;67(3):220-229. doi:10.1001/archgenpsychiatry.2010.2 20194822

[43] Mannan M, Mamun A, Doi S, Clavarino A. Is there a bi-directional relationship between depression and obesity among adult men and women? systematic review and bias-adjusted meta analysis. Asian J Psychiatr. 2016;21:51-66. doi:10.1016/j.ajp.2015.12.008 27208458

[44] Rugulies R. Depression as a predictor for coronary heart disease: a review and meta-analysis. Am J Prev Med. 2002;23(1):51-61. doi:10.1016/S0749-3797(02)00439-7 12093424

[45] Tang B, Yuan S, Xiong Y, He Q, Larsson SC. Major depressive disorder and cardiometabolic diseases: a bidirectional Mendelian randomisation study. Diabetologia. 2020;63(7):1305-1311. doi:10.1007/s00125-020-05131-6 32270255 PMC7286869

[46] Gold SM, Köhler-Forsberg O, Moss-Morris R, . Comorbid depression in medical diseases. Nat Rev Dis Primers. 2020;6(1):69. doi:10.1038/s41572-020-0200-2 32820163

[47] Pollok J, van Agteren JE, Carson-Chahhoud KV. Pharmacological interventions for the treatment of depression in chronic obstructive pulmonary disease. Cochrane Database Syst Rev. 2018;12(12):CD012346. doi:10.1002/14651858.CD012346.pub2 30566235 PMC6517114

[48] Rehm J, Gmel GE Sr, Gmel G, . The relationship between different dimensions of alcohol use and the burden of disease: an update. Addiction. 2017;112(6):968-1001. doi:10.1111/add.13757 28220587 PMC5434904

[49] Fang H, Tu S, Sheng J, Shao A. Depression in sleep disturbance: a review on a bidirectional relationship, mechanisms and treatment. J Cell Mol Med. 2019;23(4):2324-2332. doi:10.1111/jcmm.14170 30734486 PMC6433686

[50] Delgado PL. Common pathways of depression and pain. J Clin Psychiatry. 2004;65(suppl 12):16-19. 15315473

[51] Milaneschi Y, Lamers F, Berk M, Penninx BWJH. Depression heterogeneity and its biological underpinnings: toward immunometabolic depression. Biol Psychiatry. 2020;88(5):369-380. doi:10.1016/j.biopsych.2020.01.014 32247527

[52] Pitharouli MC, Hagenaars SP, Glanville KP, . Elevated C-reactive protein in patients with depression, independent of genetic, health, and psychosocial factors: results from the UK Biobank. Am J Psychiatry. 2021;178(6):522-529. doi:10.1176/appi.ajp.2020.20060947 33985349

[53] Milaneschi Y, Simmons WK, van Rossum EFC, Penninx BW. Depression and obesity: evidence of shared biological mechanisms. Mol Psychiatry. 2019;24(1):18-33. doi:10.1038/s41380-018-0017-5 29453413

[54] Frank P, Jokela M, Batty GD, Cadar D, Steptoe A, Kivimäki M. Association between systemic inflammation and individual symptoms of depression: a pooled analysis of 15 population-based cohort studies. Am J Psychiatry. 2021;178(12):1107-1118. doi:10.1176/appi.ajp.2021.20121776 34645276

[55] Frank P, Jokela M, Batty GD, Lassale C, Steptoe A, Kivimäki M. Overweight, obesity, and individual symptoms of depression: a multicohort study with replication in UK Biobank. Brain Behav Immun. 2022;105:192-200. doi:10.1016/j.bbi.2022.07.009 35853559 PMC10499756

[56] McMichael AJ, Spirtas R, Kupper LL. An epidemiologic study of mortality within a cohort of rubber workers, 1964-72. J Occup Med. 1974;16(7):458-464. 4842655

