# Supplementary material for: Association Between Depression and Physical Conditions Requiring Hospitalization
Source: JAMA Psychiatry. 2023 May 3;80(7):690–9. doi: 10.1001/jamapsychiatry.2023.0777 (PMC10157511; doi:10.1001/jamapsychiatry.2023.0777)
Supplement: Supplement 1. — eTable 1. Test of the Cox proportional hazards assumptions (PHQ-9) eTable 2. Test of the Cox proportional hazards assumptions (UKB severe MDD) eTable 3. Test of the Cox proportional hazards assumptions (UKB moderate MDD) eTable 4. Test of the Cox proportional hazards assumptions (UKB single MDD episode) eTable 5. Test of the Cox proportional hazards assumptions (UKB bipolar depression) eMethods. Statistical Code STATA. Loop for Cox proportional hazards regression analyses (example, UK Biobank) eTable 6. Baseline characteristics of included versus excluded participants (primary cohort) eTable 7. Multivariable-adjusted associations between measures of depression and the incidence of 77 diseases (UK Biobank and Finnish cohorts) eTable 8. Multivariable-adjusted associations between measures of depression and the incidence of 77 diseases (UK Biobank) eTable 9. Multivariable-adjusted associations between measures of depression and the incidence of 77 diseases (Finnish cohorts) eTable 10. Multivariable-adjusted associations between depression (PHQ-9, cut-off 10 or higher to indicate depression) and incident disease (UK Biobank) eTable 11. Multivariable-adjusted associations between one standard deviation increment in the original PHQ-9 score and incident disease (UK Biobank) eResults 1. Hazard ratios for the association of severe/moderately severe depression (PHQ-9) with incident diseases (robust depression-disease associations) in UK Biobank before and after multiple imputation eTable 12. Multivariable-adjusted associations between severe/moderately severe depression and incident disease after multiple imputation (UK Biobank) eResults 2. The association of depression with disease progression in UK Biobank participants with prevalent heart problems or diabetes eTable 13. Multivariable-adjusted Association of 77 health conditions with depression risk (Finnish Public Sector study) eTable 14. 4-year cumulative incidence per 1000 persons for 8 disease categories among people w [file jamapsychiatry-e230777-s001.pdf]

Supplemental Online Content

Frank P, Batty GD, Pentti J, et al. Association between depression and physical conditions requiring hospitalization. *JAMA Psychiatry*. Published online May 3, 2023. doi:10.1001/jamapsychiatry.2023.0777

eTable 1. Test of the Cox proportional hazards assumptions (PHQ-9)

eTable 2. Test of the Cox proportional hazards assumptions (UKB severe MDD)

eTable 3. Test of the Cox proportional hazards assumptions (UKB moderate MDD)

eTable 4. Test of the Cox proportional hazards assumptions (UKB single MDD episode)

eTable 5. Test of the Cox proportional hazards assumptions (UKB bipolar depression)

eMethods. Statistical Code STATA. Loop for Cox proportional hazards regression analyses (example, UK Biobank)

eTable 6. Baseline characteristics of included versus excluded participants (primary cohort)

eTable 7. Multivariable-adjusted associations between measures of depression and the incidence of 77 diseases (UK Biobank and Finnish cohorts)

eTable 8. Multivariable-adjusted associations between measures of depression and the incidence of 77 diseases (UK Biobank)

eTable 9. Multivariable-adjusted associations between measures of depression and the incidence of 77 diseases (Finnish cohorts)

eTable 10. Multivariable-adjusted associations between depression (PHQ-9, cut-off 10 or higher to indicate depression) and incident disease (UK Biobank)

eTable 11. Multivariable-adjusted associations between one standard deviation increment in the original PHQ-9 score and incident disease (UK Biobank)

eResults 1. Hazard ratios for the association of severe/moderately severe depression (PHQ-9) with incident diseases (robust depression-disease associations) in UK Biobank before and after multiple imputation

eTable 12. Multivariable-adjusted associations between severe/moderately severe depression and incident disease after multiple imputation (UK Biobank)

eResults 2. The association of depression with disease progression in UK Biobank participants with prevalent heart problems or diabetes

eTable 13. Multivariable-adjusted Association of 77 health conditions with depression risk (Finnish Public Sector study)

eTable 14. 4-year cumulative incidence per 1000 persons for 8 disease categories among people with and without depression (subtypes)

© 2023 Frank P et al. *JAMA Psychiatry.*

eTable 15. Hazard ratios for the association of 8 depression measures with incident disease (UK Biobank and Finnish cohorts)

eTable 16. Frequencies and cases per depression measure

eAppendix. STROBE Statement

This supplemental material has been provided by the authors to give readers additional information about their work.

© 2023 Frank P et al. *JAMA Psychiatry.*

eTable 1. **Test of the Cox proportional hazards assumptions (PHQ-9)**

Disease outcome	N (total)	N (cases)	ChiSq (df=13)	ProbChiSq
**Infections**	**127812**	**2306**	**16.72**	**0.2125**
Bacterial infections	128764	1935	16.02	0.248
Viral infections	129781	297	12.12	0.5175
**Cancer**	**118585**	**7611**	**27.53**	**0.0105**
Colorectal cancer	129663	688	12.50	0.487
Lung cancer	130503	377	13.72	0.3938
Melanoma	126529	2566	12.28	0.505
Breast cancer (women)	68470	1014	18.194	0.1099
Prostate cancer (men)	57334	1208	28.11	0.0053
Kidney cancer	130440	179	14.88	0.3148
Brain cancer	130629	104	11.43	0.4923
Leukaemia, lymphoma	129874	543	18.41	0.1426
**Diseases of the blood**	**127605**	**1748**	**18.81**	**0.1292**
Anaemia	128261	1517	15.70	0.2657
**Endocrine diseases**	**128607**	**1032**	**18.17**	**0.1511**
Diabetes	130370	121	7.04	0.8999
Obesity requiring hospital treatment	130521	39	13.69	0.3957
**Mental and behavioural disorders**	**129818**	**311**	**8.74**	**0.7925**
Dementia	130635	51	19.40	0.1112
Disorders due to substance abuse	130496	41	14.40	0.346
Mood disorders	130316	60	13.52	0.4087
Neurotic disorders	130408	84	12.28	0.5048
Psychotic disorders	130581	26	18.98	0.1238
**Diseases of the nervous system**	**124404**	**2068**	**33.51**	**0.0014**
Parkinson disease	130618	64	21.20	0.0314
Multiple sclerosis	130485	29	10.88	0.5396
Epilepsy	130488	62	17.29	0.1863
Headaches	130148	220	18.18	0.1509
TIA	130110	317	17.23	0.189
Sleep disorders	129675	143	16.45	0.2254
**Diseases of the eye**	**120519**	**6759**	**11.38**	**0.5792**
**Diseases of the ear**	**128946**	**455**	**13.46**	**0.413**
**Diseases of the circulatory system**	**113538**	**6491**	**7.85**	**0.8531**
Hypertension	130374	231	13.26	0.4283
Ischemic heart diseases	125319	2350	8.20	0.8303
Angina pectoris	128940	502	14.46	0.342
Myocardial infarction	128951	927	11.42	0.5758
Pulmonary embolism	129985	485	13.19	0.4332
Arrhythmias	127748	1591	8.42	0.8155
Heart failure	130441	341	19.95	0.0964
Stroke	129717	867	11.63	0.5579
Cerebrovascular diseases	129496	998	10.28	0.671
Intracerebral haemorrhage	130548	126	15.45	0.2178
Cerebral infarction	130112	635	9.44	0.7387
Arteriosclerosis	130534	72	14.11	0.3661
Deep vein thrombosis	129925	371	7.86	0.8525
**Diseases of the respiratory system**	**123832**	**3145**	**7.21**	**0.8913**
Influenza and pneumonia	129268	1422	7.14	0.8949
Chronic obstructive bronchitis	130334	308	19.47	0.1094
Asthma	130151	150	11.91	0.5346

© 2023 Frank P et al. *JAMA Psychiatry.*

eTable 1 continued from previous page

Disease outcome	N (total)	N (cases)	ChiSq (df=13)	ProbChiSq
**Diseases of the digestive system**	**94145**	**12938**	**24.51**	**0.0267**
Appendicitis	129708	224	9.87	0.7047
Inflammatory bowel disease	127218	911	10.59	0.6454
Diseases of liver	130288	226	13.93	0.3789
Alcoholic liver disease	130618	24	9.70	0.7181
Pancreatitis	130264	153	24.19	0.0294
**Diseases of the skin**	**121628**	**2916**	**21.49**	**0.0638**
Skin infections and eczema	128240	841	6.27	0.9358
**Diseases of the musculoskeletal system**	**107530**	**8426**	**13.03**	**0.4459**
Rheumatoid arthritis and related disorders	129676	394	17.14	0.193
Gout	130575	45	5.05	0.974
Osteoarthritis	123177	3846	23.96	0.0315
Sciatica	128842	513	20.82	0.0765
Back pain	128330	838	8.11	0.8362
Soft tissue disorders	122657	2325	16.88	0.2047
**Diseases of the genitourinary system**	**107107**	**4883**	**21.32**	**0.0669**
Renal failure	130356	372	9.68	0.72
**Pregnancy complications**	**69250**	**2**	**6.6546**	**0.8263**
Spontaneous abortion	71043	1	.	.
Hypertension in pregnancy	71266	1	.	.
Diabetes in pregnancy	71529	.	.	.
**Miscellaneous**				
Circulatory and respiratory symptoms	120954	3295	10.91	0.6183
Digestive and abdominal symptoms	119650	3937	13.36	0.4201
Injury	120539	3848	23.88	0.0323
Poisoning	130021	131	25.84	0.0179
Road accidents	130652	.	.	.
Falls	130651	13	14.50	0.3398

© 2023 Frank P et al. *JAMA Psychiatry.*

eTable 2 **Test of the Cox proportional hazards assumptions (UKB severe MDD)**

Disease outcome	N (total)	N (cases)	ChiSq (df=12)	ProbChiSq
**Infections**	**78894**	**984**	**19.2436**	**0.0828**
Bacterial infections	79228	792	18.512	0.101
Viral infections	79225	155	10.0962	0.6075
**Cancer**	**76313**	**3744**	**5.2351**	**0.9497**
Colorectal cancer	79215	368	10.8812	0.5391
Lung cancer	79500	233	12.2344	0.427
Melanoma	78635	1133	11.7756	0.4639
Breast cancer (women)	37686	581	20.8631	0.0348
Prostate cancer (men)	40474	628	9.0409	0.6181
Kidney cancer	79462	87	14.9058	0.2466
Brain cancer	79516	53	13.0584	0.3648
Leukaemia, lymphoma	79339	259	14.9701	0.2431
**Diseases of the blood**	**78638**	**979**	**17.439**	**0.1338**
Anaemia	78879	791	11.766	0.4646
**Endocrine diseases**	**78715**	**589**	**27.4868**	**0.0066**
Diabetes	79341	120	14.7513	0.2553
Obesity requiring hospital treatment	79503	52	6.8435	0.8678
**Mental and behavioural disorders**	**78960**	**269**	**14.351**	**0.2789**
Dementia	79525	38	13.5086	0.3332
Disorders due to substance abuse	79403	55	17.1468	0.1442
Mood disorders	79293	83	7.6661	0.8107
Neurotic disorders	79396	87	17.2097	0.1419
Psychotic disorders	79443	28	9.8973	0.625
**Diseases of the nervous system**	**77250**	**1502**	**6.5912**	**0.8834**
Parkinson disease	79523	28	7.9372	0.79
Multiple sclerosis	79464	25	10.1036	0.5211
Epilepsy	79427	76	9.9127	0.6236
Headaches	79383	93	16.5439	0.1676
TIA	79343	180	9.0917	0.6951
Sleep disorders	79188	186	6.7695	0.8725
**Diseases of the eye**	**76635**	**2781**	**19.2245**	**0.0833**
**Diseases of the ear**	**78928**	**363**	**14.2237**	**0.2866**
**Diseases of the circulatory system**	**72869**	**4273**	**9.3771**	**0.6704**
Hypertension	79396	97	13.9047	0.3068
Ischemic heart diseases	77104	1608	14.0254	0.2991
Angina pectoris	78515	459	5.3057	0.947
Myocardial infarction	78789	486	8.1286	0.775
Pulmonary embolism	79330	215	10.4428	0.5772
Arrhythmias	78655	789	14.4919	0.2704
Heart failure	79449	134	14.7321	0.2564
Stroke	79171	392	12.8881	0.3772
Cerebrovascular diseases	79084	460	11.9347	0.4509
Intracerebral haemorrhage	79505	68	12.9986	0.3691
Cerebral infarction	79340	274	4.1496	0.9806
Arteriosclerosis	79475	68	8.5994	0.7367
Deep vein thrombosis	79174	226	10.4788	0.574
**Diseases of the respiratory system**	**76932**	**1734**	**14.3905**	**0.2765**
Influenza and pneumonia	79093	542	5.7898	0.9263
Chronic obstructive bronchitis	79378	206	20.1142	0.065
Asthma	79289	115	10.1009	0.6071

© 2023 Frank P et al. *JAMA Psychiatry.*

eTable 2 continued from previous page

Disease outcome	N (total)	N (cases)	ChiSq (df=12)	ProbChiSq
**Diseases of the digestive system**	**67272**	**8501**	**14.7218**	**0.257**
Appendicitis	79147	156	14.0853	0.2953
Inflammatory bowel disease	78224	660	19.1643	0.0846
Diseases of liver	79371	132	8.0393	0.7821
Alcoholic liver disease	79500	26	13.8736	0.3089
Pancreatitis	79399	113	7.8206	0.799
**Diseases of the skin**	**75984**	**1709**	**5.3645**	**0.9447**
Skin infections and eczema	78597	593	10.1692	0.6011
**Diseases of the musculoskeletal system**	**72139**	**5873**	**16.0256**	**0.1901**
Rheumatoid arthritis and related disorders	79197	264	14.2683	0.2839
Gout	79515	22	9.6084	0.5659
Osteoarthritis	77324	2166	15.2935	0.2258
Sciatica	78918	491	10.5106	0.5713
Back pain	78570	577	6.2353	0.9038
Soft tissue disorders	76803	2083	7.1407	0.8482
**Diseases of the genitourinary system**	**70600**	**3883**	**10.1135**	**0.606**
Renal failure	79428	136	11.2121	0.5108
**Pregnancy complications**	**37358**	**56**	**8.3544**	**0.6813**
Spontaneous abortion	38319	19	13.9143	0.1769
Hypertension in pregnancy	38488	2	4.4044	0.221
Diabetes in pregnancy	38611	.	.	.
**Miscellaneous**				
Circulatory and respiratory symptoms	75841	2315	7.8273	0.7985
Digestive and abdominal symptoms	75546	2479	8.1287	0.775
Injury	75811	2243	18.732	0.0952
Poisoning	79140	126	17.1823	0.1429
Road accidents	79537	1	.	.
Falls	79537	11	17.4479	0.065

© 2023 Frank P et al. *JAMA Psychiatry.*

eTable 3. **Test of the Cox proportional hazards assumptions (UKB moderate MDD)**

Disease outcome	N (total)	N (cases)	ChiSq (df=12)	ProbChiSq
**Infections**	**84084**	**1058**	**27.8866**	**0.0057**
Bacterial infections	84424	850	21.7156	0.0408
Viral infections	84450	172	11.8457	0.4581
**Cancer**	**81364**	**3963**	**6.458**	**0.8913**
Colorectal cancer	84442	390	12.2944	0.4223
Lung cancer	84725	230	26.2888	0.0098
Melanoma	83824	1216	13.0805	0.3632
Breast cancer (women)	42004	645	14.8081	0.1915
Prostate cancer (men)	41280	628	10.2657	0.5067
Kidney cancer	84692	96	11.9312	0.4512
Brain cancer	84745	56	9.9102	0.6238
Leukaemia, lymphoma	84572	275	16.8594	0.155
**Diseases of the blood**	**83841**	**1030**	**16.3069**	**0.1776**
Anaemia	84084	833	12.7634	0.3865
**Endocrine diseases**	**83907**	**637**	**15.5622**	**0.2121**
Diabetes	84557	125	24.1652	0.0193
Obesity requiring hospital treatment	84732	60	3.2174	0.9938
**Mental and behavioural disorders**	**84516**	**190**	**26.6522**	**0.0087**
Dementia	84760	36	9.8671	0.5424
Disorders due to substance abuse	84681	37	12.116	0.4364
Mood disorders	84730	33	17.1673	0.1434
Neurotic disorders	84689	63	17.4564	0.1332
Psychotic disorders	84743	19	13.2492	0.3512
**Diseases of the nervous system**	**82355**	**1594**	**9.1236**	**0.6923**
Parkinson disease	84754	30	7.6747	0.7421
Multiple sclerosis	84691	28	8.9045	0.6307
Epilepsy	84665	78	11.2414	0.5084
Headaches	84598	115	10.105	0.6067
TIA	84564	180	10.6893	0.5557
Sleep disorders	84414	204	14.3422	0.2794
**Diseases of the eye**	**81724**	**2922**	**15.5924**	**0.2106**
**Diseases of the ear**	**84093**	**383**	**12.0249**	**0.4437**
**Diseases of the circulatory system**	**77715**	**4482**	**15.4445**	**0.218**
Hypertension	84622	102	12.2828	0.4232
Ischemic heart diseases	82226	1679	16.6844	0.1619
Angina pectoris	83685	443	5.9474	0.9187
Myocardial infarction	83978	511	19.9812	0.0674
Pulmonary embolism	84555	220	5.4517	0.9412
Arrhythmias	83819	834	12.6102	0.398
Heart failure	84685	142	11.0246	0.5268
Stroke	84396	404	12.3755	0.416
Cerebrovascular diseases	84304	476	10.4105	0.58
Intracerebral haemorrhage	84732	68	19.217	0.0834
Cerebral infarction	84577	280	6.2113	0.9051
Arteriosclerosis	84706	67	8.2008	0.7693
Deep vein thrombosis	84391	248	14.2626	0.2843
**Diseases of the respiratory system**	**82014**	**1786**	**14.3161**	**0.281**
Influenza and pneumonia	84313	547	11.3309	0.5008
Chronic obstructive bronchitis	84614	193	15.5675	0.2119
Asthma	84508	108	7.0085	0.857

© 2023 Frank P et al. *JAMA Psychiatry.*

eTable 3 continued from previous page

Disease outcome	N (total)	N (cases)	ChiSq (df=12)	ProbChiSq
**Diseases of the digestive system**	**71632**	**9015**	**16.3616**	**0.1752**
Appendicitis	84349	168	12.6436	0.3955
Inflammatory bowel disease	83337	695	16.6066	0.165
Diseases of liver	84610	141	6.8122	0.8698
Alcoholic liver disease	84739	24	15.7504	0.2029
Pancreatitis	84628	115	13.7108	0.3196
**Diseases of the skin**	**81015**	**1813**	**4.3347**	**0.9766**
Skin infections and eczema	83822	599	12.1304	0.4353
**Diseases of the musculoskeletal system**	**76810**	**6273**	**21.0553**	**0.0496**
Rheumatoid arthritis and related disorders	84425	277	15.0092	0.2409
Gout	84741	24	14.7397	0.256
Osteoarthritis	82396	2324	16.6376	0.1637
Sciatica	84135	535	9.3147	0.6758
Back pain	83736	618	9.4247	0.6663
Soft tissue disorders	81856	2216	7.2667	0.8395
**Diseases of the genitourinary system**	**74924**	**4177**	**11.268**	**0.5061**
Renal failure	84655	138	15.9286	0.1945
**Pregnancy complications**	**41628**	**64**	**14.4186**	**0.2107**
Spontaneous abortion	42714	19	13.1396	0.216
Hypertension in pregnancy	42869	3	6.1433	0.5231
Diabetes in pregnancy	43026	.	.	.
**Miscellaneous**				
Circulatory and respiratory symptoms	80851	2475	9.6938	0.6428
Digestive and abdominal symptoms	80500	2676	7.6887	0.809
Injury	80936	2345	15.0123	0.2408
Poisoning	84587	98	16.5104	0.169
Road accidents	84768	1	.	.
Falls	84768	9	13.2149	0.1531

© 2023 Frank P et al. *JAMA Psychiatry.*

eTable 4. **Test of the Cox proportional hazards assumptions (UKB single MDD episode)**

Disease outcome	N (total)	N (cases)	ChiSq (df=12)	ProbChiSq
**Infections**	**77937**	**908**	**21.1531**	**0.0482**
Bacterial infections	78231	730	22.8054	0.0294
Viral infections	78253	141	10.4477	0.5767
**Cancer**	**75364**	**3738**	**8.404**	**0.7528**
Colorectal cancer	78209	374	10.9008	0.5374
Lung cancer	78485	223	7.5952	0.8159
Melanoma	77637	1149	17.1607	0.1437
Breast cancer (women)	37491	582	13.5667	0.2579
Prostate cancer (men)	39669	624	9.362	0.5885
Kidney cancer	78445	90	17.545	0.1302
Brain cancer	78504	57	14.8254	0.2511
Leukaemia, lymphoma	78347	262	17.6655	0.1262
**Diseases of the blood**	**77687**	**953**	**17.2659**	**0.1399**
Anaemia	77901	767	13.4443	0.3376
**Endocrine diseases**	**77753**	**543**	**18.3875**	**0.1044**
Diabetes	78331	106	19.4565	0.0781
Obesity requiring hospital treatment	78499	40	5.7242	0.9293
**Mental and behavioural disorders**	**78277**	**170**	**16.3799**	**0.1744**
Dementia	78514	34	10.1822	0.6
Disorders due to substance abuse	78449	33	16.0468	0.1891
Mood disorders	78476	29	11.3562	0.4987
Neurotic disorders	78454	52	8.7357	0.7253
Psychotic disorders	78488	19	20.1817	0.0637
**Diseases of the nervous system**	**76440**	**1428**	**5.232**	**0.9498**
Parkinson disease	78512	29	11.3548	0.414
Multiple sclerosis	78456	23	8.6184	0.5687
Epilepsy	78428	72	11.4131	0.4939
Headaches	78389	85	13.8388	0.3111
TIA	78351	168	8.6557	0.732
Sleep disorders	78219	164	7.7911	0.8012
**Diseases of the eye**	**75728**	**2750**	**12.2847**	**0.4231**
**Diseases of the ear**	**77941**	**347**	**10.9731**	**0.5312**
**Diseases of the circulatory system**	**72020**	**4139**	**12.3841**	**0.4153**
Hypertension	78385	95	6.7815	0.8717
Ischemic heart diseases	76172	1555	17.6803	0.1257
Angina pectoris	77557	419	7.335	0.8347
Myocardial infarction	77795	476	12.5098	0.4057
Pulmonary embolism	78329	205	6.3972	0.8948
Arrhythmias	77635	790	9.16	0.6892
Heart failure	78439	129	15.9687	0.1927
Stroke	78172	372	10.9412	0.534
Cerebrovascular diseases	78092	433	6.7934	0.871
Intracerebral haemorrhage	78494	63	19.3225	0.081
Cerebral infarction	78340	265	4.6341	0.9691
Arteriosclerosis	78464	61	9.2487	0.6816
Deep vein thrombosis	78170	219	13.2822	0.3489
**Diseases of the respiratory system**	**76064**	**1623**	**12.6225**	**0.3971**
Influenza and pneumonia	78118	494	7.7418	0.805
Chronic obstructive bronchitis	78384	182	9.9935	0.6165
Asthma	78300	101	8.422	0.7513

© 2023 Frank P et al. *JAMA Psychiatry.*

eTable 4 continued from previous page

Disease outcome	N (total)	N (cases)	ChiSq (df=12)	ProbChiSq
**Diseases of the digestive system**	**66654**	**8207**	**11.3191**	**0.5018**
Appendicitis	78152	154	9.0248	0.7008
Inflammatory bowel disease	77285	591	13.7646	0.316
Diseases of liver	78387	122	10.9228	0.5355
Alcoholic liver disease	78495	21	20.896	0.0345
Pancreatitis	78400	105	4.8538	0.9627
**Diseases of the skin**	**75100**	**1657**	**8.0243**	**0.7832**
Skin infections and eczema	77648	541	7.9624	0.7881
**Diseases of the musculoskeletal system**	**71380**	**5681**	**19.7763**	**0.0714**
Rheumatoid arthritis and related disorders	78208	251	10.5521	0.5676
Gout	78501	19	11.3252	0.3328
Osteoarthritis	76352	2110	24.1149	0.0196
Sciatica	77967	443	10.2814	0.5913
Back pain	77685	521	8.8218	0.7181
Soft tissue disorders	75895	2012	4.2766	0.9779
**Diseases of the genitourinary system**	**69805**	**3777**	**12.4365**	**0.4113**
Renal failure	78423	125	9.7451	0.6383
**Pregnancy complications**	**37202**	**60**	**12.28**	**0.343**
Spontaneous abortion	38136	20	11.9241	0.2902
Hypertension in pregnancy	38277	2	4.4044	0.9273
Diabetes in pregnancy	38407	.	.	.
**Miscellaneous**				
Circulatory and respiratory symptoms	75048	2187	7.3485	0.8337
Digestive and abdominal symptoms	74829	2337	5.3212	0.9464
Injury	74985	2117	13.3986	0.3407
Poisoning	78370	77	20.1449	0.0644
Road accidents	78523	1	.	.
Falls	78523	9	16.4663	0.087

© 2023 Frank P et al. *JAMA Psychiatry.*

eTable 5. **Test of the Cox proportional hazards assumptions (UKB bipolar depression)**

Disease outcome	N (total)	N (cases)	ChiSq (df=12)	ProbChiSq
**Infections**	**72597**	**846**	**18.3738**	**0.1048**
Bacterial infections	72874	679	14.6832	0.2592
Viral infections	72886	133	12.1866	0.4308
**Cancer**	**70236**	**3476**	**8.9922**	**0.7036**
Colorectal cancer	72853	348	12.7016	0.3911
Lung cancer	73110	206	12.264	0.4247
Melanoma	72320	1069	12.1127	0.4367
Breast cancer (women)	34043	524	13.2168	0.2794
Prostate cancer (men)	72741	592	9.4225	0.583
Kidney cancer	37868	85	17.4237	0.1343
Brain cancer	73126	52	12.6045	0.32
Leukaemia, lymphoma	72973	245	15.8183	0.1997
**Diseases of the blood**	**72359**	**877**	**15.1361**	**0.2341**
Anaemia	72564	709	9.5544	0.655
**Endocrine diseases**	**72426**	**513**	**20.0468**	**0.0662**
Diabetes	72965	108	19.0827	0.0866
Obesity requiring hospital treatment	73126	32	6.243	0.9033
**Mental and behavioural disorders**	**72855**	**173**	**13.4513**	**0.3371**
Dementia	73137	32	10.5262	0.5699
Disorders due to substance abuse	73066	34	10.9568	0.5326
Mood disorders	73042	37	7.8948	0.7933
Neurotic disorders	73081	51	7.8423	0.7973
Psychotic disorders	73107	20	13.5974	0.3272
**Diseases of the nervous system**	**71202**	**1316**	**9.2048**	**0.6853**
Parkinson disease	73134	26	11.6644	0.3894
Multiple sclerosis	73082	21	10.7904	0.3741
Epilepsy	73055	65	11.2113	0.5109
Headaches	73023	82	14.8559	0.2494
TIA	72978	156	8.9946	0.7034
Sleep disorders	72856	161	6.1339	0.9092
**Diseases of the eye**	**70496**	**2547**	**17.9612**	**0.1169**
**Diseases of the ear**	**72597**	**324**	**14.0483**	**0.2976**
**Diseases of the circulatory system**	**67113**	**3872**	**12.404**	**0.4138**
Hypertension	73019	88	10.5449	0.5683
Ischemic heart diseases	70930	1455	15.2282	0.2292
Angina pectoris	72236	389	3.78	0.9871
Myocardial infarction	72454	443	10.5895	0.5644
Pulmonary embolism	72965	195	5.2245	0.9501
Arrhythmias	72320	726	13.6018	0.3269
Heart failure	73066	126	19.0408	0.0875
Stroke	72823	352	11.5994	0.4784
Cerebrovascular diseases	72743	411	7.738	0.8053
Intracerebral haemorrhage	73117	61	20.2643	0.0622
Cerebral infarction	72972	252	4.6017	0.97
Arteriosclerosis	73092	57	6.7531	0.8735
Deep vein thrombosis	72819	209	11.7592	0.4652
**Diseases of the respiratory system**	**70878**	**1539**	**13.2544**	**0.3508**
Influenza and pneumonia	72762	475	8.6089	0.7359
Chronic obstructive bronchitis	73013	177	14.8891	0.2476
Asthma	72939	95	12.7084	0.3906

© 2023 Frank P et al. *JAMA Psychiatry.*

eTable 5 continued from previous page

Disease outcome	N (total)	N (cases)	ChiSq (df=12)	ProbChiSq
**Diseases of the digestive system**	**62164**	**7624**	**14.1041**	**0.2941**
Appendicitis	72807	144	12.9261	0.3744
Inflammatory bowel disease	72019	563	13.383	0.3418
Diseases of liver	73011	115	7.6079	0.815
Alcoholic liver disease	73119	19	19.2141	0.0574
Pancreatitis	73032	97	7.7067	0.8076
**Diseases of the skin**	**69991**	**1528**	**5.6932**	**0.9308**
Skin infections and eczema	72344	511	7.3253	0.8354
**Diseases of the musculoskeletal system**	**66534**	**5260**	**16.8676**	**0.1546**
Rheumatoid arthritis and related disorders	72853	229	11.5802	0.48
Gout	73124	20	10.8254	0.458
Osteoarthritis	71145	1958	16.7791	0.1581
Sciatica	72623	409	8.6826	0.7298
Back pain	72357	478	8.545	0.7412
Soft tissue disorders	70734	1880	4.3134	0.9771
**Diseases of the genitourinary system**	**65237**	**3471**	**11.1352**	**0.5174**
Renal failure	73051	118	11.6169	0.4769
**Pregnancy complications**	**33776**	**53**	**8.1214**	**0.7024**
Spontaneous abortion	34602	19	15.0523	0.1302
Hypertension in pregnancy	34747	2	4.4044	0.221
Diabetes in pregnancy	34853	.	.	.
**Miscellaneous**				
Circulatory and respiratory symptoms	69936	2027	11.9845	0.4469
Digestive and abdominal symptoms	69728	2163	12.0616	0.4407
Injury	69846	2000	16.1753	0.1833
Poisoning	72992	75	24.5568	0.0171
Road accidents	73145	1	.	.
Falls	73145	7	12.1341	0.3536

© 2023 Frank P et al. *JAMA Psychiatry.*

***eMethods. Statistical Code STATA.* Loop for Cox proportional hazards regression analyses (example, UK Biobank)**

```
capture program drop prog_getpred
program define prog_getpred
	args row outcome title
	matrix B=r(table)
	local b=round(B[1,2],.01)
	local lci=round(B[5,2],.01)
	local uci=round(B[6,2],.01)
	local p = B[4,2]
	local string "`b' (`lci' to `uci')"
	local b2=round(B[1,3],.01)
	local lci2=round(B[5,3],.01)
	local uci2=round(B[6,3],.01)
	local p2 =  B[4,3]
	local string2 "`b2' (`lci2' to `uci2')"
	putexcel A`row'="`outcome'"
	putexcel B`row'="`title'"
	putexcel C`row'="`b'"
	putexcel D`row'="`lci'"
	putexcel E`row'="`uci'"
	putexcel F`row'="`p'"
	putexcel G`row'="`string'"
	putexcel I`row'="`b2'"
	putexcel J`row'="`lci2'"
	putexcel K`row'="`uci2'"
	putexcel L`row'="`p2'"
	putexcel M`row'="`string2'"
end
putexcel set "UKB_ICD10_PHQ9.xlsx", modify sheet("Sheet1", replace)

local covariates i.w1sex w1age_MQ i.ethnicity_origin i.education i.smoke_status i.alc_BL
i.ph_activity
local outcomes  Infections_ICD10 ///
				BacInfections_ICD10 ///
				ViralInfections_ICD10 ///
				Cancer_ICD10 ///
				ColCancer_ICD10 ///
				LungCancer_ICD10 ///
				MelanomaCancer_ICD10 ///
				BreastCancer_ICD10 ///
				ProstateCancer_ICD10 ///
				KidneyCancer_ICD10 ///
				BrainCancer_ICD10 ///
				LeukaemiaCancer_ICD10 ///
				Blood_ICD10 ///
				Anaemias_ICD10 ///
				Endocrine_ICD10 ///
				Diabetes_ICD10 ///
				Obesity_ICD10 ///
				Mental_ICD10 ///
				Dementia_ICD10 ///
```

© 2023 Frank P et al. *JAMA Psychiatry.*

Substance_ICD10 ///
Mood_ICD10 ///
Neurotic_ICD10 ///
Psychotic_ICD10 ///
NS_ICD10 ///
Parkinson_ICD10 ///
MS_ICD10 ///
Epilepsy_ICD10 ///
Headaches_ICD10 ///
TIA_ICD10 ///
Sleep_ICD10 ///
Eye_ICD10 ///
Ear_ICD10 ///
Circulatory_ICD10 ///
Hypertension_ICD10 ///
IschemicH_ICD10 ///
Angina_ICD10 ///
Myocardial_ICD10 ///
Pulmonary_ICD10 ///
Arrhythmias_ICD10 ///
HeartFailure_ICD10 ///
Stroke_ICD10 ///
Cerebrovascular_ICD10 ///
Intracerebral_ICD10 ///
CerebralInfarction_ICD10 ///
Arteriosclerosis_ICD10 ///
DeepVein_ICD10 ///
Respiratory_ICD10 ///
Influenzia_ICD10 ///
Obstructive_ICD10 ///
Asthma_ICD10 ///
Digestive_ICD10 ///
Appendicitis_ICD10 ///
IBS_ICD10 ///
Liver_ICD10 ///
AlcLiver_ICD10 ///
Pancreatitis_ICD10 ///
Skin_ICD10 ///
SkinInfect_ICD10 ///
Musculoskeletal_ICD10 ///
Rheumatoid_ICD10 ///
Gout_ICD10 ///
Osteoarthritis_ICD10 ///
Sciatica_ICD10 ///
BackPain_ICD10 ///
SoftTissue_ICD10 ///
Genitourinary_ICD10 ///
RenalFailure_ICD10 ///
Pregnancy_ICD10 ///
Abortion_ICD10 ///
HypertensionPreg_ICD10 ///
DiabetesPreg_ICD10 ///

© 2023 Frank P et al. *JAMA Psychiatry.*

```
                                        CircRespSymp_ICD10 ///
                                        DigestiveSymp_ICD10 ///
                                        Injury_ICD10 ///
                                        Poisoning_ICD10 ///
                                        RoadA_ICD10 ///
                                        Falls_ICD10
local exposure   i.PHQ9_BL3
local row=0
local num=0

foreach outcome of local outcomes {
          local row=`row'+1
stset date_`outcome', failure(`outcome'==1) id(n_eid) origin(date_mentalQ) scale(365.25)
stcox `exposure' `covariates'

prog_getpred `row' `outcome'  "Age- and sex-adjusted"

          }
import excel using "UKB_ICD10_PHQ9.xlsx", sheet(Sheet1) clear
reshape wide C, i(B) j(A) string
rename C* *
rename B covariates
```

© 2023 Frank P et al. *JAMA Psychiatry.*

	Analytical sample without missing data	Excluded participants	p-value
Participants (n)	130,652	22,916	
Age (years, SD)	63.32 (7.76)	64.36 (7.52)	< 0.001
Sex (%)			< 0.001
Men	45.22	33.46	
Women	54.78	66.54	
Ethnic origin (%)			0.449
White	97.16	97.07	
Non-white	2.84	2.93	
Education (%, n)			< 0.001
None/Elementary	6.12	11.36	
Secondary	46.43	52.91	
Tertiary	47.45	35.73	
Smoking (%, n)			< 0.001
Never	57.47	58.45	
Previous	35.36	34.29	
Current	7.18	7.25	
Alcohol (%, n)			< 0.001
None	14.02	18.38	
Low	35.52	37.67	
Moderate	26.59	23.59	
High	23.87	20.37	
Physically inactive (%, n)			0.067
Yes	46.52	46.51	
No	53.48	53.49	
Depression (%)			< 0.001
None	79.79	78.09	
Mild/Moderate	18.25	19.7	
Moderately severe/severe	1.96	2.21	

*The PHQ-9 sample from UK Biobank

***eTable 6*. Baseline characteristics of included versus excluded participants (primary cohort)**

© 2023 Frank P et al. *JAMA Psychiatry.*

eTable 7. Multivariable-adjusted associations between measures of depression and the incidence of 77 diseases (UK Biobank and Finnish cohorts)

Disease outcome	Hazard ratio (95% CI)* by exposure			
	Recurrent MD [severe] (UKB-definition)	Severe/moderately severe depression (PHQ-9)	Recurrent MD [moderate] (UKB-definition)	Mild to moderate depression (PHQ-9)
**Infections**	**1.82 (1.54 to 2.17)**	**2.36 (1.89 to 2.94)**	**1.65 (1.42 to 1.91)**	**1.51 (1.37 to 1.67)**
Bacterial infections	**1.88 (1.55 to 2.27)**	**2.52 (1.99 to 3.19)**	**1.65 (1.40 to 1.94)**	**1.53 (1.37 to 1.70)**
Viral infections	1.72 (1.12 to 2.64)	0.84 (0.35 to 2.06)	**1.91 (1.34 to 2.72)**	1.26 (0.96 to 1.67)
**Cancer**	0.95 (0.84 to 1.06)	1.05 (0.88 to 1.25)	0.99 (0.90 to 1.09)	1.05 (0.99 to 1.12)
Colorectal cancer	0.86 (0.58 to 1.29)	0.48 (0.20 to 1.15)	1.02 (0.75 to 1.39)	1.08 (0.88 to 1.31)
Lung cancer	1.37 (0.94 to 1.99)	1.37 (0.70 to 2.68)	0.88 (0.59 to 1.29)	**1.68 (1.34 to 2.12)**
Melanoma	0.75 (0.59 to 0.95)	1.20 (0.88 to 1.63)	0.99 (0.83 to 1.17)	0.93 (0.83 to 1.04)
Breast cancer (women)	1.13 (0.88 to 1.46)	0.93 (0.59 to 1.47)	1.08 (0.90 to 1.31)	1.04 (0.89 to 1.21)
Prostate cancer (men)	0.98 (0.72 to 1.34)	0.37 (0.17 to 0.83)	0.80 (0.59 to 1.10)	0.95 (0.81 to 1.13)
Kidney cancer	0.51 (0.19 to 1.40)	1.71 (0.70 to 4.22)	1.16 (0.64 to 2.11)	1.07 (0.72 to 1.58)
Brain cancer	0.17 (0.02 to 1.21)	–	0.45 (0.16 to 1.24)	0.85 (0.49 to 1.47)
Leukaemia, lymphoma	0.82 (0.50 to 1.32)	1.19 (0.61 to 2.31)	1.00 (0.69 to 1.43)	1.27 (1.02 to 1.57)
**Diseases of the blood**	1.34 (1.11 to 1.63)	**1.99 (1.51 to 2.62)**	1.19 (1.00 to 1.40)	**1.57 (1.40 to 1.76)**
Anaemia	1.34 (1.08 to 1.67)	**2.01 (1.50 to 2.70)**	1.20 (1.00 to 1.44)	**1.58 (1.40 to 1.78)**
**Endocrine diseases**	**1.65 (1.32 to 2.08)**	**2.51 (1.83 to 3.44)**	**1.61 (1.33 to 1.96)**	**1.65 (1.43 to 1.91)**
Diabetes	1.55 (0.92 to 2.62)	**5.15 (2.52 to 10.5)**	1.53 (0.96 to 2.45)	**2.49 (1.67 to 3.70)**
Obesity requiring hospital treatment	**4.86 (2.74 to 8.62)**	**6.97 (2.75 to 17.65)**	**4.21 (2.49 to 7.10)**	2.81 (1.39 to 5.68)
**Mental and behavioural disorders**	**7.79 (6.08 to 9.98)**	**5.97 (3.97 to 8.99)**	1.75 (1.23 to 2.48)	**1.76 (1.35 to 2.29)**
Dementia	2.27 (0.94 to 5.48)	6.74 (2.03 to 22.41)	1.03 (0.36 to 2.95)	2.37 (1.26 to 4.44)
Disorders due to substance abuse	**6.75 (3.88 to 11.72)**	4.11 (1.19 to 14.12)	1.68 (0.72 to 3.89)	2.16 (1.09 to 4.28)
Mood disorders	**40.78 (23.07 to 72.09)**	**5.29 (2.17 to 12.86)**	**7.10 (3.49 to 14.46)**	1.16 (0.61 to 2.23)
Neurotic disorders	**6.87 (4.44 to 10.63)**	**5.10 (2.26 to 11.53)**	1.54 (0.85 to 2.78)	2.23 (1.39 to 3.60)
Psychotic disorders	**6.54 (2.99 to 14.29)**	2.88 (0.64 to 12.88)	1.09 (0.31 to 3.82)	0.55 (0.16 to 1.85)
**Diseases of the nervous system**	**1.77 (1.54 to 2.04)**	**2.88 (2.31 to 3.59)**	**1.52 (1.34 to 1.72)**	**1.68 (1.52 to 1.86)**
Parkinson disease	1.45 (0.43 to 4.83)	**16.47 (6.77 to 40.06)**	1.66 (0.63 to 4.41)	**5.16 (3.06 to 8.72)**
Multiple sclerosis	2.03 (0.75 to 5.49)	5.48 (1.76 to 17.07)	1.64 (0.71 to 3.77)	1.57 (0.67 to 3.68)
Epilepsy	1.50 (0.78 to 2.88)	2.88 (0.87 to 9.52)	1.22 (0.66 to 2.23)	1.96 (1.12 to 3.45)
Headaches	2.06 (1.24 to 3.43)	**3.67 (2.16 to 6.25)**	**2.70 (1.82 to 3.99)**	1.69 (1.25 to 2.29)
TIA	**2.24 (1.52 to 3.31)**	2.60 (1.41 to 4.80)	1.50 (1.01 to 2.22)	1.15 (0.86 to 1.55)
Sleep disorders	**2.27 (1.56 to 3.29)**	**5.97 (3.27 to 10.89)**	**2.50 (1.81 to 3.44)**	**2.10 (1.44 to 3.06)**
**Diseases of the eye**	1.19 (1.05 to 1.35)	**1.52 (1.28 to 1.81)**	1.11 (1.00 to 1.23)	1.17 (1.09 to 1.24)
**Diseases of the ear**	1.35 (0.99 to 1.85)	**2.67 (1.70 to 4.18)**	1.24 (0.94 to 1.62)	1.09 (0.85 to 1.39)

© 2023 Frank P et al. *JAMA Psychiatry.*

eTable 7 continued from previous page

Disease outcome	Recurrent MD [severe] (UKB-definition)	Severe/moderately severe depression (PHQ-9)	Recurrent MD [moderate] (UKB-definition)	Mild to moderate depression (PHQ-9)
**Diseases of the circulatory system**	1.36 (1.24 to 1.50)	**1.95 (1.67 to 2.28)**	1.28 (1.18 to 1.39)	1.26 (1.19 to 1.35)
Hypertension	1.37 (0.74 to 2.53)	1.47 (0.60 to 3.62)	1.30 (0.77 to 2.22)	1.68 (1.24 to 2.27)
Ischemic heart diseases	**1.41 (1.21 to 1.65)**	**1.76 (1.36 to 2.29)**	1.39 (1.21 to 1.59)	1.24 (1.11 to 1.38)
Angina pectoris	**2.28 (1.78 to 2.91)**	2.20 (1.33 to 3.66)	1.27 (0.97 to 1.66)	1.21 (0.97 to 1.52)
Myocardial infarction	1.24 (0.93 to 1.66)	1.57 (1.03 to 2.39)	1.39 (1.08 to 1.77)	1.14 (0.96 to 1.36)
Pulmonary embolism	1.40 (0.92 to 2.14)	2.05 (1.25 to 3.35)	1.08 (0.73 to 1.60)	1.16 (0.92 to 1.46)
Arrhythmias	1.16 (0.91 to 1.46)	1.57 (1.11 to 2.21)	1.22 (1.00 to 1.48)	1.18 (1.03 to 1.34)
Heart failure	1.39 (0.820 to 2.36)	**4.38 (2.66 to 7.23)**	1.65 (1.06 to 2.59)	**1.98 (1.54 to 2.53)**
Stroke	1.43 (1.04 to 1.95)	1.24 (0.73 to 2.11)	1.27 (0.95 to 1.68)	1.34 (1.13 to 1.58)
Cerebrovascular diseases	1.54 (1.16 to 2.04)	1.23 (0.75 to 2.03)	1.38 (1.07 to 1.77)	1.30 (1.11 to 1.53)
Intracerebral haemorrhage	1.57 (0.75 to 3.32)	–	1.07 (0.51 to 2.27)	1.26 (0.80 to 1.98)
Cerebral infarction	1.17 (0.78 to 1.76)	1.36 (0.74 to 2.48)	1.04 (0.71 to 1.50)	1.41 (1.16 to 1.71)
Arteriosclerosis	2.38 (1.29 to 4.42)	1.27 (0.30 to 5.29)	1.70 (0.90 to 3.23)	0.67 (0.33 to 1.35)
Deep vein thrombosis	1.20 (0.79 to 1.83)	2.28 (1.32 to 3.93)	1.57 (1.13 to 2.17)	1.25 (0.97 to 1.62)
**Diseases of the respiratory system**	1.48 (1.29 to 1.71)	**2.33 (1.91 to 2.83)**	1.18 (1.04 to 1.35)	**1.54 (1.41 to 1.67)**
Influenza and pneumonia	**1.58 (1.24 to 2.01)**	**2.65 (2.01 to 3.50)**	1.15 (0.91 to 1.46)	**1.53 (1.35 to 1.74)**
Chronic obstructive bronchitis	1.78 (1.24 to 2.56)	**4.11 (2.56 to 6.60)**	0.92 (0.59 to 1.42)	**2.08 (1.62 to 2.67)**
Asthma	2.16 (1.36 to 3.43)	2.01 (0.81 to 5.00)	0.95 (0.56 to 1.60)	1.68 (1.16 to 2.43)
**Diseases of the digestive system**	1.44 (1.35 to 1.54)	**1.67 (1.49 to 1.87)**	1.31 (1.24 to 1.38)	1.38 (1.32 to 1.44)
Appendicitis	0.83 (0.48 to 1.45)	1.82 (0.88 to 3.73)	0.93 (0.61 to 1.42)	0.96 (0.68 to 1.36)
Inflammatory bowel disease	**2.05 (1.67 to 2.51)**	1.81 (1.24 to 2.63)	**1.60 (1.33 to 1.93)**	1.48 (1.27 to 1.73)
Diseases of liver	1.79 (1.12 to 2.86)	2.30 (1.20 to 4.42)	1.79 (1.19 to 2.69)	1.59 (1.17 to 2.16)
Alcoholic liver disease	**4.86 (2.1 to 11.24)**	1.53 (0.19 to 12.16)	3.49 (1.42 to 8.59)	2.64 (1.13 to 6.15)
Pancreatitis	1.58 (0.93 to 2.67)	1.43 (0.52 to 3.94)	1.13 (0.68 to 1.86)	**1.89 (1.33 to 2.69)**
**Diseases of the skin**	1.42 (1.23 to 1.64)	1.09 (0.83 to 1.45)	1.27 (1.12 to 1.44)	1.11 (1.01 to 1.22)
Skin infections and eczema	**1.77 (1.41 to 2.22)**	**2.01 (1.36 to 2.97)**	1.21 (0.98 to 1.51)	1.45 (1.23 to 1.71)
**Diseases of the musculoskeletal system**	1.44 (1.33 to 1.56)	**1.99 (1.74 to 2.28)**	1.32 (1.23 to 1.41)	1.43 (1.36 to 1.51)
Rheumatoid arthritis and related disorders	1.56 (1.10 to 2.21)	**2.54 (1.50 to 4.30)**	1.24 (0.91 to 1.69)	**1.57 (1.25 to 1.99)**
Gout	2.06 (0.60 to 7.05)	**6.80 (2.32 to 19.93)**	2.22 (0.81 to 6.04)	1.78 (0.86 to 3.66)
Osteoarthritis	1.35 (1.18 to 1.54)	**1.80 (1.46 to 2.20)**	1.32 (1.18 to 1.47)	1.36 (1.26 to 1.47)
Sciatica	**2.17 (1.73 to 2.73)**	**2.73 (1.78 to 4.19)**	**1.98 (1.62 to 2.41)**	**1.90 (1.56 to 2.31)**
Back pain	**2.09 (1.69 to 2.58)**	**3.99 (2.96 to 5.38)**	**1.76 (1.46 to 2.12)**	**2.10 (1.81 to 2.45)**
Soft tissue disorders	1.42 (1.24 to 1.62)	**1.81 (1.41 to 2.32)**	1.31 (1.17 to 1.46)	1.34 (1.21 to 1.48)

© 2023 Frank P et al. *JAMA Psychiatry.*

eTable 7 continued from previous page

Disease outcome	Recurrent MD [severe] (UKB-definition)	Severe/moderately severe depression (PHQ-9)	Recurrent MD [moderate] (UKB-definition)	Mild to moderate depression (PHQ-9)
**Diseases of the genitourinary system**	1.40 (1.27 to 1.54)	**1.87 (1.58 to 2.22)**	1.30 (1.20 to 1.41)	1.32 (1.23 to 1.42)
Renal failure	1.81 (1.12 to 2.93)	**3.66 (2.30 to 5.83)**	1.37 (0.86 to 2.18)	**1.77 (1.40 to 2.25)**
**Pregnancy complications**	0.59 (0.23 to 1.48)	–	0.84 (0.45 to 1.56)	3.31 (0.19 to 58.40)
Spontaneous abortion	0.30 (0.04 to 2.32)	–	0.19 (0.03 to 1.43)	–
Hypertension in pregnancy	–	–	2.08 (0.18 to 23.97)	–
Diabetes in pregnancy	–	–	–	–
**Miscellaneous**				
Circulatory and respiratory symptoms	**1.76 (1.56 to 1.97)**	**2.16 (1.78 to 2.62)**	**1.64 (1.48 to 1.81)**	**1.50 (1.38 to 1.63)**
Digestive and abdominal symptoms	**1.71 (1.53 to 1.91)**	**2.15 (1.80 to 2.57)**	**1.51 (1.37 to 1.66)**	**1.53 (1.42 to 1.65)**
Injury	1.44 (1.27 to 1.63)	**1.86 (1.54 to 2.26)**	1.16 (1.04 to 1.29)	1.28 (1.18 to 1.38)
Poisoning	**8.43 (5.87 to 12.1)**	**8.63 (5.04 to 14.76)**	**2.72 (1.77 to 4.18)**	1.98 (1.33 to 2.96)
Road accidents	–	–	–	–
Falls	6.19 (1.75 to 21.89)	**23.03 (4.58 to 115.85)**	2.35 (0.47 to 11.71)	3.55 (1.03 to 12.30)
Self-harm	–	–	–	–

*Hazard ratio for depression as predictor of disease adjusted for age, sex, education, ethnicity, smoking, alcohol, and physical activity at baseline.

© 2023 Frank P et al. *JAMA Psychiatry.*

eTable 8. Multivariable-adjusted associations between measures of depression and the incidence of 77 diseases (UK Biobank)

Disease outcome	Hazard ratio (95% CI)* by exposure			
	Single MD episode (UKB-definition)	Bipolar depression (UKB-definition)	Inflammation-related depression (PHQ-9)	Obesity-related depression (PHQ-9)
**Infections**	1.14 (0.91 to 1.43)	1.58 (1.04 to 2.39)	2.00 (1.68 to 2.37)	1.96 (1.6 to 2.39)
Bacterial infections	1.17 (0.91 to 1.50)	1.65 (1.04 to 2.61)	2.11 (1.76 to 2.54)	2.11 (1.71 to 2.61)
Viral infections	1.00 (0.55 to 1.82)	1.63 (0.60 to 4.43)	1.17 (0.67 to 2.06)	0.62 (0.26 to 1.51)
**Cancer**	1.04 (0.92 to 1.16)	0.85 (0.63 to 1.13)	1.16 (1.02 to 1.31)	0.98 (0.84 to 1.15)
Colorectal cancer	1.18 (0.82 to 1.70)	1.18 (0.53 to 2.65)	0.97 (0.61 to 1.54)	0.86 (0.49 to 1.53)
Lung cancer	1.28 (0.83 to 1.98)	1.34 (0.59 to 3.03)	1.42 (0.90 to 2.24)	1.10 (0.60 to 2.01)
Melanoma	0.98 (0.79 to 1.22)	0.64 (0.34 to 1.20)	0.89 (0.69 to 1.16)	0.83 (0.61 to 1.14)
Breast cancer (women)	1.04 (0.80 to 1.35)	0.67 (0.30 to 1.50)	1.22 (0.91 to 1.65)	0.95 (0.64 to 1.42)
Prostate cancer (men)	1.06 (0.77 to 1.47)	0.85 (0.40 to 1.79)	0.59 (0.36 to 0.95)	0.61 (0.35 to 1.05)
Kidney cancer	1.06 (0.49 to 2.30)	1.50 (0.37 to 6.13)	0.89 (0.36 to 2.19)	1.29 (0.53 to 3.15)
Brain cancer	1.08 (0.43 to 2.72)	–	0.69 (0.17 to 2.82)	–
Leukaemia, lymphoma	1.07 (0.68 to 1.67)	1.12 (0.41 to 3.01)	1.29 (0.81 to 2.05)	1.13 (0.63 to 2.00)
**Diseases of the blood**	1.25 (1.01 to 1.55)	1.20 (0.75 to 1.91)	1.94 (1.58 to 2.36)	1.67 (1.30 to 2.13)
Anaemia	1.23 (0.96 to 1.56)	1.30 (0.79 to 2.14)	1.97 (1.59 to 2.44)	1.79 (1.38 to 2.31)
**Endocrine diseases**	1.01 (0.74 to 1.37)	1.55 (0.93 to 2.61)	2.33 (1.84 to 2.95)	1.71 (1.26 to 2.33)
Diabetes	0.40 (0.13 to 1.25)	2.48 (1.00 to 6.14)	3.61 (2.03 to 6.42)	2.60 (1.25 to 5.40)
Obesity requiring hospital treatment	3.09 (1.49 to 6.42)	2.05 (0.48 to 8.70)	4.83 (2.33 to 10.04)	4.57 (2.04 to 10.22)
**Mental and behavioural disorders**	1.84 (1.18 to 2.87)	9.34 (6.09 to 14.35)	3.39 (2.35 to 4.90)	3.91 (2.63 to 5.81)
Dementia	0.89 (0.21 to 3.73)	–	3.69 (1.30 to 10.43)	2.48 (0.59 to 10.32)
Disorders due to substance abuse	1.27 (0.38 to 4.19)	4.67 (1.61 to 13.57)	3.30 (1.25 to 8.69)	3.44 (1.19 to 9.93)
Mood disorders	11.03 (5.22 to 23.33)	66.07 (33.26 to 131.23)	2.73 (1.15 to 6.50)	3.93 (1.66 to 9.32)
Neurotic disorders	0.82 (0.30 to 2.30)	3.28 (1.00 to 10.71)	2.13 (0.97 to 4.70)	3.09 (1.40 to 6.80)
Psychotic disorders	2.28 (0.65 to 8.00)	10.61 (3.39 to 33.18)	2.88 (0.83 to 9.99)	2.61 (0.60 to 11.36)
**Diseases of the nervous system**	1.41 (1.20 to 1.67)	2.09 (1.55 to 2.81)	2.27 (1.91 to 2.71)	2.26 (1.85 to 2.77)
Parkinson disease	2.24 (0.77 to 6.51)	3.02 (0.40 to 22.59)	4.16 (1.65 to 10.5)	7.26 (3.10 to 17.01)
Multiple sclerosis	1.28 (0.38 to 4.33)	2.63 (0.35 to 19.83)	2.72 (0.92 to 8.06)	5.26 (1.95 to 14.24)
Epilepsy	1.30 (0.59 to 2.85)	–	1.32 (0.41 to 4.28)	1.21 (0.29 to 4.99)
Headaches	1.59 (0.86 to 2.96)	5.36 (2.64 to 10.90)	2.85 (1.86 to 4.37)	2.83 (1.73 to 4.63)
TIA	1.64 (1.03 to 2.63)	3.56 (1.74 to 7.32)	1.83 (1.08 to 3.10)	1.67 (0.89 to 3.15)
Sleep disorders	1.29 (0.74 to 2.24)	3.36 (1.81 to 6.25)	3.39 (1.98 to 5.79)	3.32 (1.82 to 6.07)
**Diseases of the eye**	1.20 (1.05 to 1.37)	1.22 (0.91 to 1.63)	1.29 (1.13 to 1.47)	1.38 (1.19 to 1.61)
**Diseases of the ear**	1.07 (0.74 to 1.57)	1.24 (0.58 to 2.63)	1.54 (1.01 to 2.36)	1.80 (1.13 to 2.86)

© 2023 Frank P et al. *JAMA Psychiatry.*

eTable 8 continued from previous page

Disease outcome	Single MD episode (UKB-definition)	Bipolar depression (UKB-definition)	Inflammation-related depression (PHQ-9)	Obesity-related depression (PHQ-9)
**Diseases of the circulatory system**	1.18 (1.06 to 1.32)	1.49 (1.22 to 1.83)	1.61 (1.43 to 1.82)	1.53 (1.32 to 1.77)
Hypertension	1.41 (0.73 to 2.72)	2.13 (0.67 to 6.81)	2.02 (1.17 to 3.50)	1.78 (0.91 to 3.48)
Ischemic heart diseases	1.25 (1.04 to 1.49)	1.40 (0.99 to 1.97)	1.41 (1.14 to 1.75)	1.31 (1.02 to 1.69)
Angina pectoris	1.43 (1.03 to 1.98)	1.72 (0.94 to 3.14)	1.85 (1.24 to 2.76)	1.68 (1.03 to 2.74)
Myocardial infarction	1.28 (0.93 to 1.76)	1.16 (0.60 to 2.26)	1.35 (0.96 to 1.88)	1.06 (0.68 to 1.63)
Pulmonary embolism	0.97 (0.57 to 1.64)	1.63 (0.67 to 3.99)	1.73 (1.17 to 2.57)	1.80 (1.14 to 2.82)
Arrhythmias	1.36 (1.07 to 1.72)	1.29 (0.76 to 2.19)	1.45 (1.12 to 1.89)	1.16 (0.83 to 1.63)
Heart failure	1.26 (0.68 to 2.35)	3.92 (1.90 to 8.09)	3.24 (2.19 to 4.81)	2.53 (1.52 to 4.20)
Stroke	0.98 (0.66 to 1.47)	1.09 (0.48 to 2.45)	1.33 (0.92 to 1.92)	1.37 (0.89 to 2.10)
Cerebrovascular diseases	1.00 (0.69 to 1.44)	1.40 (0.72 to 2.71)	1.28 (0.90 to 1.81)	1.41 (0.96 to 2.09)
Intracerebral haemorrhage	0.68 (0.21 to 2.18)	1.17 (0.16 to 8.53)	0.34 (0.05 to 2.46)	–
Cerebral infarction	0.92 (0.56 to 1.51)	1.02 (0.38 to 2.75)	1.47 (0.98 to 2.23)	1.46 (0.90 to 2.37)
Arteriosclerosis	1.46 (0.63 to 3.42)	1.96 (0.47 to 8.12)	1.92 (0.76 to 4.86)	1.07 (0.26 to 4.42)
Deep vein thrombosis	1.05 (0.64 to 1.70)	2.16 (1.06 to 4.41)	1.52 (0.94 to 2.45)	1.53 (0.88 to 2.68)
**Diseases of the respiratory system**	0.96 (0.80 to 1.16)	1.36 (0.98 to 1.87)	1.98 (1.70 to 2.30)	1.78 (1.49 to 2.14)
Influenza and pneumonia	0.81 (0.56 to 1.17)	1.36 (0.77 to 2.43)	2.26 (1.83 to 2.80)	2.17 (1.68 to 2.79)
Chronic obstructive bronchitis	0.93 (0.53 to 1.65)	2.16 (1.05 to 4.43)	3.31 (2.31 to 4.73)	2.79 (1.79 to 4.35)
Asthma	1.16 (0.60 to 2.24)	2.23 (0.81 to 6.13)	2.65 (1.48 to 4.73)	1.62 (0.71 to 3.69)
**Diseases of the digestive system**	1.14 (1.05 to 1.23)	1.18 (1.00 to 1.38)	1.56 (1.43 to 1.70)	1.43 (1.29 to 1.58)
Appendicitis	0.87 (0.48 to 1.57)	0.68 (0.17 to 2.75)	1.69 (0.96 to 2.99)	2.00 (1.09 to 3.70)
Inflammatory bowel disease	0.99 (0.74 to 1.33)	2.10 (1.34 to 3.30)	1.75 (1.33 to 2.31)	1.54 (1.10 to 2.17)
Diseases of liver	1.28 (0.70 to 2.33)	2.34 (0.95 to 5.78)	2.23 (1.38 to 3.62)	1.93 (1.07 to 3.48)
Alcoholic liver disease	3.25 (1.08 to 9.76)	6.59 (1.48 to 29.26)	2.17 (0.62 to 7.60)	1.83 (0.41 to 8.07)
Pancreatitis	1.07 (0.54 to 2.12)	0.55 (0.08 to 3.96)	1.90 (1.02 to 3.55)	1.43 (0.63 to 3.27)
**Diseases of the skin**	1.22 (1.04 to 1.44)	1.31 (0.92 to 1.85)	1.13 (0.92 to 1.38)	0.96 (0.75 to 1.24)
Skin infections and eczema	0.96 (0.70 to 1.32)	1.30 (0.73 to 2.31)	1.99 (1.49 to 2.64)	1.39 (0.94 to 2.05)
**Diseases of the musculoskeletal system**	1.17 (1.07 to 1.28)	1.32 (1.10 to 1.59)	1.78 (1.61 to 1.96)	1.60 (1.42 to 1.81)
Rheumatoid arthritis and related disorders	1.19 (0.79 to 1.79)	1.02 (0.38 to 2.75)	2.76 (1.92 to 3.97)	2.30 (1.46 to 3.62)
Gout	–	3.36 (0.44 to 25.54)	4.25 (1.63 to 11.09)	4.33 (1.51 to 12.40)
Osteoarthritis	1.15 (0.99 to 1.34)	1.34 (0.97 to 1.84)	1.55 (1.33 to 1.82)	1.50 (1.25 to 1.81)
Sciatica	1.34 (0.99 to 1.81)	1.81 (1.06 to 3.10)	2.62 (1.92 to 3.57)	1.75 (1.15 to 2.68)
Back pain	1.25 (0.94 to 1.65)	1.30 (0.73 to 2.31)	3.08 (2.45 to 3.88)	2.80 (2.13 to 3.69)
Soft tissue disorders	1.18 (1.01 to 1.37)	1.76 (1.35 to 2.31)	1.67 (1.38 to 2.02)	1.42 (1.12 to 1.79)

© 2023 Frank P et al. *JAMA Psychiatry.*

eTable 8 continued from previous page

Disease outcome	Single MD episode (UKB-definition)	Bipolar depression (UKB-definition)	Inflammation-related depression (PHQ-9)	Obesity-related depression (PHQ-9)
**Diseases of the genitourinary system**	1.23 (1.11 to 1.37)	1.25 (0.99 to 1.57)	1.52 (1.32 to 1.74)	1.61 (1.38 to 1.88)
Renal failure	1.02 (0.51 to 2.01)	1.03 (0.25 to 4.20)	2.42 (1.63 to 3.58)	2.26 (1.42 to 3.61)
**Pregnancy complications**	1.28 (0.63 to 2.62)	1.74 (0.42 to 7.21)	–	–
Spontaneous abortion	0.78 (0.18 to 3.41)	2.30 (0.30 to 17.47)	–	–
Hypertension in pregnancy	–	–	–	–
Diabetes in pregnancy	–	–	–	–
**Miscellaneous**				
Circulatory and respiratory symptoms	1.31 (1.14 to 1.51)	1.66 (1.27 to 2.17)	1.76 (1.51 to 2.05)	1.77 (1.48 to 2.11)
Digestive and abdominal symptoms	1.17 (1.02 to 1.35)	1.52 (1.17 to 1.99)	1.69 (1.47 to 1.94)	1.70 (1.44 to 2.00)
Injury	0.94 (0.80 to 1.10)	1.52 (1.16 to 2.00)	1.65 (1.42 to 1.91)	1.67 (1.41 to 1.99)
Poisoning	2.23 (1.22 to 4.09)	7.56 (3.91 to 14.62)	5.12 (3.21 to 8.18)	4.72 (2.76 to 8.08)
Road accidents	–	–	–	–
Falls	4.01 (0.82 to 19.66)	–	14.35 (3.77 to 54.59)	12.36 (2.64 to 57.89)
Self-harm	–	–	–	–

*Hazard ratio for depression as predictor of disease adjusted for age, sex, education, ethnicity, smoking, alcohol, and physical activity at baseline.

© 2023 Frank P et al. *JAMA Psychiatry.*

***eTable 9*. Multivariable-adjusted associations between measures of depression and the incidence of 77 diseases (Finnish cohorts)**

	**Hazard ratio (95% CI)* by exposure**	
**Disease outcome**	**Self-reported doctor-diagnosed depression**	**Hospitalisation due to depression**
**Infections**	1.30 (1.19 to 1.43)	2.06 (1.41 to 3.01)
Bacterial infections	1.32 (1.20 to 1.46)	2.37 (1.62 to 3.47)
Viral infections	1.28 (1.00 to 1.63)	1.49 (0.48 to 4.63)
**Cancer**	1.04 (0.97 to 1.12)	0.72 (0.45 to 1.14)
Colorectal cancer	0.74 (0.56 to 0.98)	–
Lung cancer	1.32 (0.99 to 1.75)	2.02 (0.65 to 6.33)
Melanoma	0.92 (0.78 to 1.09)	0.84 (0.32 to 2.25)
Breast cancer (women)	1.08 (0.97 to 1.21)	1.12 (0.64 to 1.98)
Prostate cancer (men)	1.41 (1.11 to 1.79)	0.60 (0.08 to 4.27)
Kidney cancer	0.88 (0.52 to 1.48)	–
Brain cancer	1.10 (0.69 to 1.78)	–
Leukaemia, lymphoma	1.06 (0.84 to 1.33)	–
**Diseases of the blood**	1.53 (1.26 to 1.85)	1.67 (0.69 to 4.03)
Anaemia	1.40 (1.09 to 1.81)	2.34 (0.87 to 6.27)
**Endocrine diseases**	1.42 (1.33 to 1.50)	1.51 (1.12 to 2.02)
Diabetes	1.38 (1.29 to 1.47)	1.33 (0.94 to 1.89)
Obesity requiring hospital treatment	2.45 (1.95t o 3.08)	0.57 (0.08 to 4.03)
**Mental and behavioural disorders**	4.39 (4.04 to 4.78)	–
Dementia	1.11 (0.87 to 1.42)	2.92 (1.21 to 7.07)
Disorders due to substance abuse	3.37 (2.89 to 3.94)	10.56 (7.12 to 15.68)
Mood disorders	5.85 (5.10 to 6.71)	12.31 (8.66 to 17.51)
Neurotic disorders	6.78 (6.04 to 7.61)	
Psychotic disorders	5.04 (4.13 to 6.16)	5.10 (2.53 to 10.31)
**Diseases of the nervous system**	1.41 (1.32 to 1.51)	1.48 (1.07 to 2.04)
Parkinson disease	1.29 (0.93 to 1.79)	–
Multiple sclerosis	1.37 (0.98 to 1.92)	2.63 (0.84 to 8.23)
Epilepsy	1.51 (1.23 to 1.85)	2.00 (0.83 to 4.82)
Headaches	1.82 (1.44 to 2.31)	1.76 (0.57 to 5.50)
TIA	1.37 (1.15 to 1.65)	0.91 (0.29 to 2.82)
Sleep disorders	2.07 (1.88 to 2.27)	2.21 (1.48 to 3.31)
**Diseases of the eye**	1.15 (1.07 to 1.24)	1.26 (0.88 to 1.82)
**Diseases of the ear**	1.37 (1.17 to 1.61)	1.84 (0.92 to 3.69)
**Diseases of the circulatory system**	1.16 (1.10 to 1.23)	1.20 (0.90 to 1.58)
Hypertension	1.12 (1.04 to 1.20)	1.20 (0.90 to 1.58)
Ischemic heart diseases	1.24 (1.12 to 1.37)	1.00 (0.57 to 1.76)
Angina pectoris	1.21 (1.04 to 1.42)	0.62 (0.20 to 1.91)
Myocardial infarction	1.23 (1.04 to 1.46)	0.96 (0.36 to 2.56)
Pulmonary embolism	1.88 (1.51 to 2.36)	1.61 (0.52 to 5.03)
Arrhythmias	1.12 (1.02 to 1.22)	1.23 (0.79 to 1.91)
Heart failure	1.30 (1.05 to 1.62)	0.43 (0.06 to 3.03)
Stroke	1.19 (1.03 to 1.39)	1.53 (0.76 to 3.06)
Cerebrovascular diseases	1.20 (1.05 to 1.37)	1.26 (0.63 to 2.52)
Intracerebral haemorrhage	1.00 (0.68 to 1.47)	–
Cerebral infarction	1.33 (1.11 to 1.59)	2.10 (1.00 to 4.43)
Arteriosclerosis	1.24 (0.90 to 1.71)	0.88 (0.12 to 6.28)
Deep vein thrombosis	1.38 (1.09 to 1.74)	1.35 (0.43 to 4.20)
**Diseases of the respiratory system**	1.40 (1.32 to 1.50)	1.65 (1.22 to 2.23)
Influenza and pneumonia	1.48 (1.33 to 1.64)	2.55 (1.73 to 3.76)
Chronic obstructive bronchitis	1.69 (1.37 to 2.07)	2.66 (1.26 to 5.63)
Asthma	1.43 (1.31 to 1.57)	1.89 (1.30 to 2.74)

© 2023 Frank P et al. *JAMA Psychiatry.*

eTable 9 continued from previous page

**Diseases of the digestive system**	1.20 (1.14 to 1.27)	1.62 (1.29 to 2.05)
Appendicitis	1.15 (0.98 to 1.34)	1.56 (0.78 to 3.12)
Inflammatory bowel disease	1.07 (0.88 to 1.30)	2.30 (1.19 to 4.45)
Diseases of liver	1.84 (1.50 to 2.27)	0.45 (0.06 to 3.23)
Alcoholic liver disease	2.51 (1.89 to 3.34)	1.06 (0.15 to 7.54)
Pancreatitis	1.72 (1.37 to 2.17)	3.15 (1.41 to 7.07)
**Diseases of the skin**	1.48 (1.28 to 1.71)	1.78 (0.93 to 3.44)
Skin infections and eczema	1.39 (1.13 to 1.72)	2.81 (1.33 to 5.92)
**Diseases of the musculoskeletal system**	1.18 (1.13 to 1.24)	1.18 (0.94 to 1.48)
Rheumatoid arthritis and related disorders	1.05 (0.93 to 1.18)	0.91 (0.47 to 1.76)
Gout	1.22 (0.86 to 1.73)	–
Osteoarthritis	1.21 (1.13 to 1.30)	1.75 (1.27 to 2.39)
Sciatica	1.19 (1.03 to 1.37)	1.70 (0.94 to 3.08)
Back pain	1.70 (1.42 to 2.03)	2.51 (1.25 to 5.04)
Soft tissue disorders	1.18 (1.09 to 1.27)	0.84 (0.53 to 1.33)
**Diseases of the genitourinary system**	1.22 (1.16 to 1.29)	1.16 (0.88 to 1.53)
Renal failure	1.24 (0.92 to 1.66)	0.69 (0.10 to 4.94)
**Pregnancy complications**	0.99 (0.86 to 1.13)	1.62 (0.90 to 2.93)
Spontaneous abortion	0.95 (0.69 to 1.30)	0.75 (0.11 to 5.31)
Hypertension in pregnancy	1.07 (0.78 to 1.46)	1.61 (0.40 to 6.47)
Diabetes in pregnancy	1.01 (0.76 to 1.34)	2.31 (0.86 to 6.20)
**Miscellaneous**		
Circulatory and respiratory symptoms	1.49 (1.33 to 1.67)	1.20 (0.65 to 2.24)
Digestive and abdominal symptoms	1.37 (1.23 to 1.53)	3.16 (2.20 to 4.53)
Injury	1.20 (1.13 to 1.27)	1.63 (1.26 to 2.11)
Poisoning	5.22 (4.38 to 6.22)	14.46 (9.84 to 21.25)
Road accidents	1.25 (1.01 to 1.54)	2.87 (1.49 to 5.56)
Falls	1.23 (1.13 to 1.34)	1.61 (1.13 to 2.30)
Self-harm	5.56 (4.53 to 6.83)	16.07 (10.67 to 24.20)

*Hazard ratio for depression as predictor of disease adjusted for age, sex, education, ethnicity, smoking, alcohol, and physical activity at baseline.

© 2023 Frank P et al. *JAMA Psychiatry.*

***eTable 10*. Multivariable-adjusted associations between depression (PHQ-9, cut-off 10 or higher to indicate depression) and incident disease (UK Biobank)**

Disease outcome	Hazard ratio (95% confidence interval)
**Infections**	1.88 (1.63 to 2.17)
Bacterial infections	1.96 (1.68 to 2.29)
Viral infections	1.21 (0.77 to 1.89)
**Cancer**	1.04 (0.93 to 1.15)
Colorectal cancer	0.91 (0.63 to 1.33)
Lung cancer	1.54 (1.06 to 2.22)
Melanoma	0.92 (0.75 to 1.13)
Kidney cancer	0.90 (0.44 to 1.85)
Brain cancer	1.06 (0.43 to 2.64)
Leukaemia, lymphoma	0.95(0.62 to 1.45)
**Diseases of the blood**	1.85 (1.57 to 2.19)
Anaemia	1.89 (1.58 to 2.25)
**Endocrine diseases**	2.08 (1.7 to 2.54)
Diabetes	3.03 (1.81 to 5.07)
Obesity requiring hospital treatment	5.88 (3.02 to 11.45)
**Mental and behavioural disorders**	2.99 (2.16 to 4.12)
Dementia	4.14 (1.83 to 9.34)
Disorders due to substance abuse	3.60 (1.61 to 8.03)
Mood disorders	2.70 (1.30 to 5.60)
Neurotic disorders	1.98 (1.01 to 3.89)
Psychotic disorders	2.54 (0.85 to 7.63)
**Diseases of the nervous system**	2.36 (2.05 to 2.71)
Parkinson disease	9.92 (5.56 to 17.72)
Multiple sclerosis	2.25 (0.84 to 6.06)
Epilepsy	2.42 (1.13 to 5.18)
Headaches	2.56 (1.76 to 3.72)
TIA	1.73 (1.12 to 2.66)
Sleep disorders	3.19 (2.01 to 5.05)
**Diseases of the eye**	1.33 (1.19 to 1.47)
**Diseases of the ear**	1.49 (1.05 to 2.11)
**Diseases of the circulatory system**	1.56 (1.41 to 1.73)
Hypertension	2.01 (1.29 to 3.15)
Ischemic heart diseases	1.45 (1.23 to 1.72)
Angina pectoris	1.78 (1.29 to 2.48)
Myocardial infarction	1.28 (0.97 to 1.68)
Pulmonary embolism	1.68 (1.21 to 2.33)
Arrhythmias	1.29 (1.04 to 1.60)
Heart failure	2.73 (1.93 to 3.87)
Stroke	1.19 (0.88 to 1.62)
Cerebrovascular diseases	1.24 (0.93 to 1.64)
Intracerebral haemorrhage	0.62 (0.20 to 1.95)
Cerebral infarction	1.31 (0.93 to 1.85)
Arteriosclerosis	1.56 (0.66 to 3.65)
Deep vein thrombosis	1.49 (1.01 to 2.20)
**Diseases of the respiratory system**	1.80 (1.58 to 2.04)
Influenza and pneumonia	1.92 (1.60 to 2.32)
Chronic obstructive bronchitis	2.71 (1.95 to 3.76)
Asthma	2.04 (1.2 to 3.45)

© 2023 Frank P et al. *JAMA Psychiatry.*

eTable 10 continued from previous page

**Diseases of the digestive system**	1.55 (1.45 to 1.67)
Appendicitis	1.19 (0.70 to 2.03)
Inflammatory bowel disease	1.70 (1.35 to 2.14)
Diseases of liver	2.18 (1.45 to 3.28)
Alcoholic liver disease	2.02 (0.66 to 6.13)
Pancreatitis	2.21 (1.35 to 3.61)
**Diseases of the skin**	1.15 (0.98 to 1.35)
Skin infections and eczema	1.86 (1.47 to 2.36)
**Diseases of the musculoskeletal system**	1.74 (1.61 to 1.89)
Rheumatoid arthritis and related disorders	2.37 (1.72 to 3.25)
Gout	3.19 (1.32 to 7.71)
Osteoarthritis	1.63 (1.44 to 1.84)
Sciatica	2.47 (1.90 to 3.21)
Back pain	2.73 (2.23 to 3.34)
Soft tissue disorders	1.51 (1.29 to 1.77)
**Diseases of the genitourinary system**	1.62 (1.46 to 1.80)
Renal failure	2.79 (2.04 to 3.80)
**Miscellaneous**	
Circulatory and respiratory symptoms	1.85 (1.64 to 2.09)
Digestive and abdominal symptoms	1.64 (1.46 to 1.84)
Injury	1.49 (1.32 to 1.69)
Poisoning	5.80 (3.89 to 8.65)
Road accidents	-
Falls	12.71 (3.79 to 42.69)
Self-harm	-

*Hazard ratio for depression as predictor of disease adjusted for age, sex, education, ethnicity, smoking, alcohol, and physical activity at baseline.

© 2023 Frank P et al. *JAMA Psychiatry.*

eTable 11. **Multivariable-adjusted associations between one standard deviation increment in the original PHQ-9 score and incident disease (UK Biobank)**

Disease outcome	Hazard ratio (95% confidence interval) per 1 standard deviation increment
**Infections**	1.23 (1.19 to 1.27)
Bacterial infections	1.24 (1.2 to 1.29)
Viral infections	1.1 (0.99 to 1.22)
**Cancer**	1.02 (0.99 to 1.04)
Colorectal cancer	0.99 (0.91 to 1.08)
Lung cancer	1.19 (1.10 to 1.30)
Melanoma	0.96 (0.92 to 1.00)
Kidney cancer	1.07 (0.93 to 1.24)
Brain cancer	0.86 (0.67 to 1.10)
Leukaemia, lymphoma	1.05 (0.97 to 1.15)
**Diseases of the blood**	1.23 (1.18 to 1.28)
Anaemia	1.23 (1.18 to 1.28)
**Endocrine diseases**	1.26 (1.20 to 1.32)
Diabetes	1.47 (1.31 to 1.64)
Obesity requiring hospital treatment	1.56 (1.33 to 1.83)
**Mental and behavioural disorders**	1.43 (1.33 to 1.55)
Dementia	1.50 (1.23 to 1.83)
Disorders due to substance abuse	1.39 (1.13 to 1.69)
Mood disorders	1.38 (1.16 to 1.64)
Neurotic disorders	1.37 (1.19 to 1.58)
Psychotic disorders	1.20 (0.89 to 1.61)
**Diseases of the nervous system**	1.30 (1.26 to 1.35)
Parkinson disease	1.88 (1.64 to 2.15)
Multiple sclerosis	1.34 (1.07 to 1.68)
Epilepsy	1.30 (1.08 to 1.56)
Headaches	1.39 (1.28 to 1.52)
TIA	1.16 (1.04 to 1.28)
Sleep disorders	1.49 (1.34 to 1.65)
**Diseases of the eye**	1.10 (1.07 to 1.13)
**Diseases of the ear**	1.14 (1.05 to 1.23)
**Diseases of the circulatory system**	1.16 (1.14 to 1.19)
Hypertension	1.25 (1.12 to 1.39)
Ischemic heart diseases	1.14 (1.10 to 1.19)
Angina pectoris	1.19 (1.11 to 1.29)
Myocardial infarction	1.10 (1.04 to 1.17)
Pulmonary embolism	1.16 (1.07 to 1.25)
Arrhythmias	1.10 (1.04 to 1.15)
Heart failure	1.39 (1.28 to 1.50)
Stroke	1.13 (1.06 to 1.20)
Cerebrovascular diseases	1.13 (1.06 to 1.20)
Intracerebral haemorrhage	1.04 (0.86 to 1.27)
Cerebral infarction	1.14 (1.06 to 1.23)
Arteriosclerosis	1.06 (0.85 to 1.32)
Deep vein thrombosis	1.16 (1.06 to 1.27)
**Diseases of the respiratory system**	1.24 (1.20 to 1.28)
Influenza and pneumonia	1.27 (1.21 to 1.32)
Chronic obstructive bronchitis	1.40 (1.30 to 1.51)
Asthma	1.23 (1.08 to 1.40)

© 2023 Frank P et al. *JAMA Psychiatry.*

eTable 11 continued from previous page

**Diseases of the digestive system**	1.17 (1.15 to 1.19)
Appendicitis	1.09 (0.97 to 1.23)
Inflammatory bowel disease	1.21 (1.14 to 1.27)
Diseases of liver	1.26 (1.14 to 1.39)
Alcoholic liver disease	1.24 (0.96 to 1.61)
Pancreatitis	1.24 (1.09 to 1.40)
**Diseases of the skin**	1.06 (1.02 to 1.10)
Skin infections and eczema	1.22 (1.15 to 1.29)
**Diseases of the musculoskeletal system**	1.20 (1.18 to 1.22)
Rheumatoid arthritis and related disorders	1.25 (1.15 to 1.35)
Gout	1.37 (1.11 to 1.70)
Osteoarthritis	1.18 (1.15 to 1.21)
Sciatica	1.28 (1.20 to 1.37)
Back pain	1.38 (1.32 to 1.45)
Soft tissue disorders	1.16 (1.12 to 1.20)
**Diseases of the genitourinary system**	1.16 (1.13 to 1.19)
Renal failure	1.35 (1.25 to 1.45)
**Miscellaneous**	
Circulatory and respiratory symptoms	1.22 (1.19 to 1.26)
Digestive and abdominal symptoms	1.22 (1.18 to 1.25)
Injury	1.15 (1.12 to 1.18)
Poisoning	1.58 (1.43 to 1.74)
Road accidents	
Falls	1.89 (1.41 to 2.54)
Self-harm	

*Hazard ratio for depression as predictor of disease adjusted for age, sex, education, ethnicity, smoking, alcohol, and physical activity at baseline.

© 2023 Frank P et al. *JAMA Psychiatry.*

eResults 1. Hazard ratios for the association of severe/moderately severe depression (PHQ-9) with incident diseases (robust depression-disease associations) in UK Biobank before and after multiple imputation

We performed a sensitivity analysis reporting the results of a multiple imputation analysis with 20 imputed datasets to account for missing data on covariates in UK Biobank. As in our primary analysis, hazard ratios (HRs) and accompanying 95% confidence intervals (CIs) were adjusted for age and sex, and, additionally, ethnic origin, education, smoking, alcohol consumption, and physical activity. Missingness of covariates was as follows:

Variable	Missing	Total	Percent Missing
Age	0	153,568	0.00
Sex	0	153,568	0.00
Ethnicity	73	153,568	0.05
Smoking	72	153,568	0.05
Alcohol	73	153,568	0.05
Physical activity	22,604	153,568	14.72
Education	1,399	153,568	0.91

The hazard ratios for the association of moderately severe/severe depression with incident diseases (robust depression disease associations) in UK Biobank before and after multiple imputation are presented in the table below.

		Severe/moderately severe depression (PHQ-9, UK Biobank) Hazard ratio (95% confidence interval)	
**Disease category**	**Diagnosis or diagnostic group***	**Non-imputed data**	**Imputed data**
**Poisoning, falls, injuries**	Poisoning	8.63 (5.04 to 14.76)	8.93 (5.49 to 14.52)
	Falls	23.03 (4.58 to 115.85)	18.48 (3.84 to 88.95)
	Injury	1.86 (1.54 to 2.26)	1.92 (1.61 to 2.28)
**Diseases of the endocrine, genitourinary, and digestive systems and symptoms**	Obesity requiring hospital treatment	6.97 (2.75 to 17.65)	10.3 (4.83 to 21.99)
	Diabetes	5.15 (2.52 to 10.50)	3.75 (1.86 to 7.58)
	Renal failure	3.66 (2.30 to 5.83)	3.57 (2.33 to 5.45)
	Digestive and abdominal symptoms	2.15 (1.80 to 2.57)	2.08 (1.77 to 2.45)
	Diseases of the digestive system	1.67 (1.49 to 1.87)	1.70 (1.53 to 1.89)
**Mental and behavioural disorders, diseases of the nervous system**	Mood disorders	5.29 (2.17 to 12.86)	7.74 (3.78 to 15.86)
	Neurotic disorders	5.10 (2.26 to 11.53)	5.04 (2.36 to 10.78)
	Sleep disorders	5.97 (3.27 to 10.89)	6.56 (3.86 to 11.16)
	Headaches	3.67 (2.16 to 6.25)	3.77 (2.34 to 6.08)
	Parkinson disease	16.47 (6.77 to 40.06)	22.49 (10.63 to 47.58)
**Diseases of the musculoskeletal system**	Back pain	3.99 (2.96 to 5.38)	4.13 (3.17 to 5.38)
	Gout‡	6.80 (2.32 to 19.93)	5.78 (2.2 to 15.22)
	Sciatica	2.73 (1.78 to 4.19)	2.78 (1.90 to 4.05)
	Rheumatoid arthritis and related disorders	2.54 (1.50 to 4.30)	2.58 (1.63 to 4.08)
	Osteoarthritis	1.80 (1.46 to 2.20)	1.78 (1.48 to 2.14)
	Soft tissue disorders	1.81 (1.41 to 2.32)	1.84 (1.48 to 2.29)
**Diseases of the respiratory system**	Chronic obstructive bronchitis	4.11 (2.56 to 6.60)	3.24 (2.03 to 5.16)
	Influenza and pneumonia	2.65 (2.01 to 3.50)	2.72 (2.12 to 3.48)
**Diseases of the circulatory system and blood and related symptoms**	Heart failure	4.38 (2.66 to 7.23)	4.13 (2.61 to 6.54)
	Circulatory and respiratory symptoms	2.16 (1.78 to 2.62)	1.97 (1.71 to 2.27)
	Anaemia	2.01 (1.50 to 2.70)	1.95 (1.49 to 2.55)
	Ischaemic heart diseases	1.76 (1.36 to 2.29)	1.79 (1.42 to 2.27)
**Diseases of the ear or eye**	Diseases of the ear	2.67 (1.70 to 4.18)	2.81 (1.89 to 4.19)
	Diseases of the eye	1.52 (1.28 to 1.81)	1.57 (1.34 to 1.83)
**Infections and diseases of the skin**	Skin infections and eczema	2.01 (1.36 to 2.97)	1.77 (1.23 to 2.55)
	Bacterial infections	2.52 (1.99 to 3.19)	2.52 (2.04 to 3.12)

†Hazard ratio for depression adjusted for age, sex, education, ethnicity, smoking, alcohol, and physical activity at baseline.

© 2023 Frank P et al. *JAMA Psychiatry.*

eTable 12. **Multivariable-adjusted associations between severe/moderately severe depression and incident disease after multiple imputation (UK Biobank)**

Disease outcome	Hazard ratio (95% confidence interval)
**Infections**	2.35 (1.92 to 2.87)
Bacterial infections	2.52 (2.04 to 3.12)
Viral infections	0.89 (0.39 to 2.00)
**Cancer**	1.10 (0.94 to 1.30)
Colorectal cancer	0.65 (0.32 to 1.30)
Lung cancer	1.33 (0.72 to 2.44)
Melanoma	1.21 (0.91 to 1.61)
Kidney cancer	1.70 (0.74 to 3.87)
Brain cancer	-
Leukaemia, lymphoma	0.98 (0.51 to 1.91)
**Diseases of the blood**	1.91 (1.49 to 2.47)
Anaemia	1.95 (1.49 to 2.55)
**Endocrine diseases**	2.76 (2.10 to 3.63)
Diabetes	3.75 (1.86 to 7.58)
Obesity requiring hospital treatment	10.3 (4.83 to 21.99)
**Mental and behavioural disorders**	6.49 (4.49 to 9.37)
Dementia	6.29 (2.23 to 17.74)
Disorders due to substance abuse	4.71 (1.59 to 13.98)
Mood disorders	7.74 (3.78 to 15.86)
Neurotic disorders	5.04 (2.36 to 10.78)
Psychotic disorders	2.43 (0.55 to 10.64)
**Diseases of the nervous system**	3.00 (2.47 to 3.65)
Parkinson disease	22.49 (10.63 to 47.58)
Multiple sclerosis	4.26 (1.40 to 12.94)
Epilepsy	3.58 (1.26 to 10.18)
Headaches	3.77 (2.34 to 6.08)
TIA	2.34 (1.30 to 4.20)
Sleep disorders	6.56 (3.86 to 11.16)
**Diseases of the eye**	1.57 (1.34 to 1.83)
**Diseases of the ear**	2.81 (1.89 to 4.19)
**Diseases of the circulatory system**	1.97 (1.71 to 2.27)
Hypertension	1.82 (0.89 to 3.72)
Ischemic heart diseases	1.79 (1.42 to 2.27)
Angina pectoris	2.30 (1.46 to 3.62)
Myocardial infarction	1.64 (1.13 to 2.37)
Pulmonary embolism	1.82 (1.14 to 2.9)
Arrhythmias	1.60 (1.17 to 2.18)
Heart failure	4.13 (2.61 to 6.54)
Stroke	1.6 (1.05 to 2.46)
Cerebrovascular diseases	1.58 (1.06 to 2.36)
Intracerebral haemorrhage	0.57 (0.08 to 4.07)
Cerebral infarction	1.71 (1.05 to 2.77)
Arteriosclerosis	2.20 (0.79 to 6.14)
Deep vein thrombosis	1.80 (1.05 to 3.10)
**Diseases of the respiratory system**	2.31 (1.94 to 2.76)
Influenza and pneumonia	2.72 (2.12 to 3.48)
Chronic obstructive bronchitis	3.24 (2.03 to 5.16)
Asthma	1.42 (0.58 to 3.5)

© 2023 Frank P et al. *JAMA Psychiatry.*

eTable 12 continued from previous page

**Diseases of the digestive system**	1.70 (1.53 to 1.89)
Appendicitis	1.81 (0.95 to 3.44)
Inflammatory bowel disease	1.83 (1.31 to 2.57)
Diseases of liver	1.90 (0.99 to 3.63)
Alcoholic liver disease	1.22 (0.16 to 9.5)
Pancreatitis	1.55 (0.68 to 3.55)
**Diseases of the skin**	1.06 (0.82 to 1.38)
Skin infections and eczema	1.77 (1.23 to 2.55)
**Diseases of the musculoskeletal system**	1.95 (1.73 to 2.2)
Rheumatoid arthritis and related disorders	2.58 (1.63 to 4.08)
Gout	5.78 (2.20 to 15.22)
Osteoarthritis	1.78 (1.48 to 2.14)
Sciatica	2.78 (1.90 to 4.05)
Back pain	4.13 (3.17 to 5.38)
Soft tissue disorders	1.84 (1.48 to 2.29)
**Diseases of the genitourinary system**	2.00 (1.72 to 2.33)
Renal failure	3.57 (2.33 to 5.45)
**Miscellaneous**	
Circulatory and respiratory symptoms	2.11 (1.77 to 2.52)
Digestive and abdominal symptoms	2.08 (1.77 to 2.45)
Injury	1.92 (1.61 to 2.28)
Poisoning	8.93 (5.49 to 14.52)
Road accidents	-
Falls	18.48 (3.84 to 88.95)
Self-harm	-

*Hazard ratio for depression as predictor of disease adjusted for age, sex, education, ethnicity, smoking, alcohol, and physical activity at baseline.

© 2023 Frank P et al. *JAMA Psychiatry.*

eResults 2. The association of depression with disease progression in UK Biobank participants with prevalent heart problems or diabetes

We conducted two additional Cox proportional hazards regression analyses for participants with prevalent cardiometabolic diseases who had not been hospitalised due to these conditions to determine whether depression was associated with an increased risk of hospitalisations at follow-up. Hazard ratios (HRs) and accompanying 95% confidence intervals (CIs) were adjusted for age and sex, and, additionally, ethnic origin, education, smoking, alcohol consumption, and physical activity. Accordingly, the first analysis included a subgroup of 23,509 participants who reported heart problems at baseline but had not been hospitalised due to circulatory conditions. Of these, 2311 were hospitalised due to circulatory conditions at follow-up, suggesting disease progression. The results show that after multivariable adjustment, participants with mild/moderate depression had a 1.26 times increased risk (95% CIs, 1.14 to 1.40), and those with severe/moderately severe depression a 1.92 times increased risk (95% CIs, 1.52 to 2.43) of hospitalisations due to circulatory conditions at follow-up relative to participants without depression at baseline. Similar results were apparent in the second analysis in which we included a subgroup of 4,161 participants with self-reported doctor-diagnosed diabetes at baseline who had not been hospitalised due to the condition. At follow-up, 65 of these participants required hospital treatment in secondary care. Relative to participants without depression at baseline, individuals with mild/moderate depression had a 2.35 times increased risk (95% CIs, 1.38 to 3.98), and those with severe/moderately severe depression a 3.60 times higher risk (95% CIs, 1.46 to 8.90) of hospitalisation due to diabetes at follow-up.

© 2023 Frank P et al. *JAMA Psychiatry.*

***eTable 13*. Multivariable-adjusted Association of 77 health conditions with depression risk (Finnish Public Sector study)**

				Self-reported depression (FPS)	
**Diseases**	**Disease status**	**N(Total)**	**N(Depr)**	**OddsRatio (95%CI)***	**p-value**
**Poisoning, falls, injuries**	No	54481	5056	1 (ref)	
	Yes	2685	263	1.11 (0.097 to 1.26)	0.1263
**Poisoning**	No	57111	5304	1 (ref)	
	Yes	55	15	3.20 (1.76 to 5.81)	0.0001
**Falls**	No	55892	5184	1 (ref)	
	Yes	1274	135	1.21 (1.01 to 1.45)	0.0404
**Injury**	No	54599	5075	1 (ref)	
	Yes	2567	244	1.08 (0.94 to 1.23)	0.2922
**Diseases of the endocrine, genitourinary and digestive systems and symptoms**	No	52743	4821	1 (ref)	
	Yes	4423	498	1.25 (1.13 to 1.38)	<0.0001
**Obesity requiring hospital treatment**	No	57148	5315	1 (ref)	
	Yes	18	4	2.33 (0.76 to 7.13)	0.1403
**Diabetes**	No	56506	5253	1 (ref)	
	Yes	660	66	1.11 (0.86 to 1.44)	0.4221
**Renal failure**	No	57134	5316	1 (ref)	
	Yes	32	3	0.98 (0.30 to 3.22)	0.9675
**Digestive and abdominal symptoms**	No	56254	5190	1 (ref)	
	Yes	912	129	1.54 (1.27 to 1.86)	<0.0001
**Diseases of the digestive system**	No	54118	4987	1 (ref)	
	Yes	3048	332	1.20 (1.07 to 1.35)	0.0023
**Mental and behavioural disorders, diseases of the nervous system**	No	56718	5242	1 (ref)	
	Yes	448	77	2.13 (1.66 to 2.74)	<0.0001
**Mood disorders**	No	57118	5301	1 (ref)	
	Yes	48	18	5.38 (2.98 to 9.70)	<0.0001
**Neurotic disorders**	No	57112	5312	1 (ref)	
	Yes	54	7	1.45 (0.65 to 3.22)	0.3633
**Sleep disorders**	No	56925	5280	1 (ref)	
	Yes	241	39	2.10 (1.48 to 2.97)	<0.0001
**Headaches**	No	57072	5307	1 (ref)	
	Yes	94	12	1.42 (0.77 to 2.61)	0.2598
**Parkinson disease**	No	57148	5317	1 (ref)	
	Yes	18	2	1.22 (0.28 to 5.32)	0.7954
**Diseases of the musculoskeletal system**	No	53657	4934	1 (ref)	
	Yes	3509	385	1.23 (1.10 to 1.37)	0.0003
**Back pain**	No	56952	5297	1 (ref)	
	Yes	214	22	1.16 (0.74 to 1.80)	0.5224
**Gout**	No	57116	5313	1 (ref)	
	Yes	50	6	1.56 (0.66 to 3.68)	0.3100
**Sciatica**	No	56611	5235	1 (ref)	
	Yes	555	84	1.77 (1.40 to 2.24)	<0.0001
**Rheumatoid arthritis and related disorders**	No	56301	5258	1 (ref)	
	Yes	865	61	0.73 (0.56 to 0.95)	0.0202
**Osteoarthritis**	No	56424	5241	1 (ref)	
	Yes	742	78	1.15 (0.91 to 1.46)	0.2518
**Soft tissue disorders**	No	55845	5149	1 (ref)	
	Yes	1321	170	1.47 (1.25 to 1.73)	<0.0001
**Diseases of the respiratory system**	No	56764	5277	1 (ref)	
	Yes	402	42	1.13 (0.82 to 1.56)	0.4541
**Chronic obstructive bronchitis**	No	57145	5315	1 (ref)	
	Yes	21	4	2.18 (0.73 to 6.53)	0.1654
**Influenza and pneumonia**	No	56783	5281	1 (ref)	
	Yes	383	38	1.07 (0.76 to 1.50)	0.7004
**Diseases of the circulatory system and blood and related symptoms**	No	56169	5204	1 (ref)	
	Yes	997	115	1.35 (1.10 to 1.64)	0.0035
**Heart failure**	No	57121	5313	1 (ref)	
	Yes	45	6	1.59 (0.67 to 3.78)	0.2936
**Circulatory and respiratory symptoms**	No	56611	5252	1 (ref)	
	Yes	555	67	1.39 (1.07 to 1.80)	0.0127
**Anaemia**	No	57099	5311	1 (ref)	
	Yes	67	8	1.28 (0.61 to 2.69)	0.5110

© 2023 Frank P et al. *JAMA Psychiatry.*

eTable 13 continued from previous page

				Self-reported depression (FPS)	
**Diseases**	**Disease status**	**N(Total)**	**N(Depr)**	**OddsRatio (95%CI)***	**p-value**
**Ischemic heart diseases**	No	56782	5279	1 (ref)	
	Yes	384	40	1.23 (0.89 to 1.72)	0.2157
**Diseases of the ear or eye**	No	56229	5236	1 (ref)	
	Yes	937	83	0.96 (0.76 to 1.20)	0.7158
**Diseases of the ear**	No	56788	5286	1 (ref)	
	Yes	378	33	0.96 (0.67 to 1.37)	0.8022
**Diseases of the eye**	No	56603	5269	1 (ref)	
	Yes	563	50	0.95 (0.71 to 1.28)	0.7533
**Infections and diseases of the skin**	No	56420	5223	1 (ref)	
	Yes	746	96	0.95 (0.71 to 1.28)	0.0009
**Skin infections and eczema**	No	56960	5295	1 (ref)	
	Yes	206	24	1.25 (0.82 to 1.92)	0.3022
**Bacterial infections**	No	56623	5247	1 (ref)	
	Yes	543	72	1.51 (1.18 to 1.94)	0.0012

*Odds ratios adjusted for age, sex, education, smoking, alcohol, and physical activity. Participants with depression at baseline were excluded from the analyses. Follow-up depression: 2 surveys, 4 and 8 years after baseline.

Disease category	Severe/moderately severe depression* Absolute risk	No Depression Absolute risk (ref.)	% Absolute excess risk
Endocrine, genitourinary, digestive	245	147	9·8%
Musculoskeletal	91	54	3·7%
Circulator and blood	86	47	3·9%
Eve and ear	60	54	0·6%
Poisoning, falls, injury	53	28	2·5%
Infections and skin	39	19	2.0%
Respiratory	23	11	1·2%
Mental, neurological, behavioural	20	3	1·7%
All 29 diseases†	500	337	16.3%

† These associations were robust to multivariable adjustment, had an HR of greater than or equal to 1.50, and were statistically significant at a Bonferroni corrected alpha level, P<0.0006.

*The PHQ-9 sample from UK Biobank

***eTable 14.* 4-year cumulative incidence per 1000 persons for 8 disease categories among people with and without depression (subtypes)**

© 2023 Frank P et al. *JAMA Psychiatry.*

Disease category	Diagnosis or diagnostic group*	Mild to moderate depression (PHQ-9)	Inflammation-related symptom profile (PHQ-9)	Obesity-related symptom profile (PHQ-9)	Recurrent severe major depression (UKB definition)
**Poisoning, falls, injuries**	Poisoning	–	5·12 (3·21 to 8·18)	4·72 (2·76 to 8·08)	8.43 (5.87 to 12.10)
	Falls	–	14·35 (3·77 to 54·59)	–	–
	Injury	–	1·65 (1·42 to 1·91)	1·67 (1·41 to 1·99)	–
**Diseases of the endocrine, genitourinary, and digestive systems and symptoms**	Obesity requiring hospital treatment	–	4·83 (2·33 to 10·04)	4·57 (2·04 to 10·22)	4.86 (2.74 to 8.62)
	Diabetes	2.49 (1.67 to 3.7)	3·61 (2·03 to 6·42)	–	–
	Renal failure‡	1.77 (1.4 to 2.25)	2·42 (1·63 to 3·58)	2·26 (1·42 to 3·61)	–
	Digestive and abdominal symptoms	1.53 (1.42 to 1.65)	1·69 (1·47 to 1·94)	1·70 (1·44 to 2·00)	–
	Diseases of the digestive system	–	1·56 (1·43 to 1·70)	–	1.71 (1.53 to 1.91)
**Mental and behavioural disorders, diseases of the nervous system**	Mood disorders	–	–	–	40.78 (23.07 to 72.09)
	Neurotic disorders	–	–	–	6.87 (4.44 to 10.63)
	Sleep disorders	2.10 (1.44 to 3.06)	3·39 (1·98 to 5·79)	3·32 (1·82 to 6·07)	2.27 (1.56 to 3.29)
	Headaches	–	2·85 (1·86 to 4·37)	2·83 (1·73 to 4·63)	–
	Parkinson disease‡	5.16 (3.06 to 8.72)	–	7·26 (3·10 to 17·01)	–
**Diseases of the musculoskeletal system**	Back pain	2.10 (1.81 to 2.45)	3·08 (2·45 to 3·88)	2·80 (2·13 to 3·69)	2.09 (1.69 to 2.58)
	Gout‡	–	–	–	–
	Sciatica	1.90 (1.56 to 2.31)	2·62 (1·92 to 3·57)	–	2.17 (1.73 to 2.73)
	Rheumatoid arthritis and related disorders‡	1.57 (1.25 to 1.99)	2·76 (1·92 to 3·97)	2·30 (1·46 to 3·62)	–
	Osteoarthritis	–	1·55 (1·33 to 1·82)	1·50 (1·25 to 1·81)	–
	Soft tissue disorders	–	1·67 (1·38 to 2·02)	–	–
**Diseases of the respiratory system**	Chronic obstructive bronchitis	2.08 (1.62 to 2.67)	3·31 (2·31 to 4·73)	2·79 (1·79 to 4·35)	–
	Influenza and pneumonia	1.53 (1.35 to 1.74)	2·26 (1·83 to 2·80)	2·17 (1·68 to 2·79)	1.58 (1.24 to 2.01)
**Diseases of the circulatory system and blood and related symptoms**	Heart failure	1.98 (1.54 to 2.53)	3·24 (2·19 to 4·81)	2·26 (1·42 to 3·61)	–
	Circulatory and respiratory symptoms	1.50 (1.38 to 1.63)	1·76 (1·51 to 2·05)	1·77 (1·48 to 2·11)	–
	Anaemia	1.58 (1.4 to 1.78)	1·97 (1·59 to 2·44)	1·79 (1·38 to 2·31)	–
	Ischaemic heart diseases	–	–	–	–
**Diseases of the ear or eye**	Diseases of the ear	–	–	–	–
	Diseases of the eye	–	–	–	–
**Infections and diseases of the skin**	Skin infections and eczema	–	1·99 (1·49 to 2·64)	–	–
	Bacterial infections	1.53 (1.37 to 1.70)	2·11 (1·76 to 2·54)	2·11 (1·71 to 2·61)	1.88 (1.55 to 2.27)

eTable 15. **Hazard ratios for the association of 8 depression measures with incident disease (UK Biobank and Finnish cohorts)**

(Continues on next page)

© 2023 Frank P et al. *JAMA Psychiatry.*

Disease category	Diagnosis or diagnostic group*	Recurrent moderate major depression (UKB definition)	Single major depression episode (UKB definition)	Bipolar depression (UKB definition)	Hospitalisation due to depression (Finnish cohorts)
(Continued from previous page)					
**Poisoning, falls, injuries**	Poisoning	2.72 (1.77 to 4.18)	–	7.56 (3.91 to 14.62)	13.54 (9.22 to 19.89)
	Falls	–	–	–	–
	Injury	–	–	–	1.58 (1.23 to 2.04)
**Diseases of the endocrine, genitourinary, and digestive systems and symptoms**	Obesity requiring hospital treatment	4.21 (2.49 to 7.10)		–	–
	Diabetes	–	–	–	–
	Renal failure‡	–	–	–	–
	Digestive and abdominal symptoms	–		–	2.97 (2.07 to 4.26)
	Diseases of the digestive system	1.51 (1.37 to 1.66)	–	–	1.67 (1.33 to 2.08)
**Mental and behavioural disorders, diseases of the nervous system**	Mood disorders	7.10 (3.49 to 14.46)	11.03 (5.22 to 23.33)	66.07 (33.26 to 131.23)	12.99 (9.31 to 18.12)
	Neurotic disorders	–	–	–	n.a.
	Sleep disorders	2.5 (1.81 to 3.44)	–	3.36 (1.81 to 6.25)	2.14 (1.44 to 3.18)
	Headaches	2.7 (1.82 to 3.99)	–	5.36 (2.64 to 10.9)	–
	Parkinson disease‡	–	–	–	n.a.
**Diseases of the musculoskeletal system**	Back pain	1.76 (1.46 to 2.12)	–	–	–
	Gout‡	–	–	–	n.a.
	Sciatica	1.98 (1.62 to 2.41)	–	–	–
	Rheumatoid arthritis and related disorders‡			–	
	Osteoarthritis	–	–	–	–
	Soft tissue disorders	–	–	1.76 (1.35 to 2.31)	–
**Diseases of the respiratory system**	Chronic obstructive bronchitis	–	–	–	–
	Influenza and pneumonia	–	–	–	2.45 (1.68 to 3.59)
**Diseases of the circulatory system and blood and related symptoms**	Heart failure	–	–	3.92 (1.90 to 8.09)	–
	Circulatory and respiratory symptoms	1.64 (1.48 to 1.81)		1.66 (1.27 to 2.17)	
	Anaemia	–	–	–	–
	Ischaemic heart diseases	–	–	–	–
**Diseases of the ear or eye**	Diseases of the ear	–	–	–	–
	Diseases of the eye	–	–	–	–
**Infections and diseases of the skin**	Skin infections and eczema	–	–	–	–
	Bacterial infections	1.65 (1.4 to 1.94)	–	–	2.31 (1.59 to 3.35)

eTable 15. **Hazard ratios for the association of 8 depression measures with incident disease (UK Biobank and Finnish cohorts)**

*Hazard ratio for depression as predictor of disease adjusted for age, sex, education, ethnicity, smoking, alcohol, and physical activity at baseline.

© 2023 Frank P et al. *JAMA Psychiatry.*

***eTable 16*. Frequencies and cases per depression measure**

Disease outcome													
**Disease category (ICD-10 chapter)**	**Diagnosis or diagnostic group***	**ICD10-codes**	**No depression**		**Mild to moderate**		**Moderately severe to severe**		**Inflammation-related symptom profile**		**Obesity-related symptom profile**		**N (total)**
			**N (cases)**	**N (total)**	**N (cases)**	**N (total)**	**N (cases)**	**N (total)**	**N (cases)**	**N (total)**	**N (cases)**	**N (total)**	
**Infections**		**A01 - B89**	**1680**	**102253**	**540**	**23105**	**86**	**2454**	**145**	**4382**	**103**	**3152**	**127812**
	Bacterial infections	A01 - A79	1406	102910	453	23367	76	2487	127	4439	92	3197	128764
	Viral infections	A80 - B34	225	103645	67	23602	5	2534	13	4524	5	3236	129781
**Cancer**		**C00 - C97**	**6202**	**94603**	**1286**	**21614**	**123**	**2368**	**250**	**4198**	**154**	**3022**	**118585**
	Colorectal cancer	C18, C20	562	103471	121	23640	5	2552	19	4548	12	3258	129663
	Lung cancer	C34	266	104142	102	23798	9	2563	20	4570	11	3275	130503
	Melanoma	C43 - C44	2149	100811	375	23202	42	2516	59	4477	39	3205	126529
	Breast cancer (women)	C50	780	53011	207	13957	19	1502	46	2757	25	1893	68470
	Prostate cancer (men)	C61	1038	47382	164	8975	6	977	17	1660	13	1269	57334
	Kidney cancer	C64	143	104075	31	23801	5	2564	5	4574	5	3276	130440
	Brain cancer	C71	89	104228	15	23836	0	2565	2	4576	0	3278	130629
	Leukaemia, lymphoma	C81 - C96	430	103639	104	23681	9	2554	19	4554	12	3266	129874
**Diseases of the blood**		**D50 - D89**	**1273**	**102025**	**421**	**23127**	**54**	**2453**	**105**	**4386**	**66**	**3150**	**127605**
	Anaemia	D50 - D64	1101	102509	368	23278	48	2474	94	4427	62	3177	128261
**Endocrine diseases**		**E00 - E35**	**726**	**102853**	**264**	**23304**	**42**	**2450**	**79**	**4399**	**43**	**3151**	**128607**
	Diabetes	E10 - E14	74	104074	38	23749	9	2547	14	4548	8	3258	130370
	Obesity requiring hospital treatment	E66	16	104191	16	23785	7	2545	11	4546	8	3256	130521
**Mental and behavioural disorders**		**F00 - F99**	**204**	**103803**	**79**	**23553**	**28**	**2462**	**34**	**4448**	**28**	**3166**	**129818**
	Dementia	F00 - F03, G30, G31	34	104232	14	23840	3	2563	4	4574	2	3275	130635
	Disorders due to substance abuse	F10 - F19	25	104162	13	23788	3	2546	5	4554	4	3258	130496
	Mood disorders	F30 - F39	42	104075	12	23736	6	2505	6	4507	6	3214	130316
	Neurotic disorders	F40 - F48	50	104114	27	23755	7	2539	7	4545	7	3251	130408
	Psychotic disorders	F20 - F29	21	104209	3	23811	2	2561	3	4566	2	3270	130581

eTable 16 continued from previous page

Disease outcome													
**Disease category (ICD-10 chapter)**	**Diagnosis or diagnostic group***	**ICD10-codes**	**No depression**		**Mild to moderate**		**Moderately severe to severe**		**Inflammation-related symptom profile**		**Obesity-related symptom profile**		**N (total)**
			**N (cases)**	**N (total)**	**N (cases)**	**N (total)**	**N (cases)**	**N (total)**	**N (cases)**	**N (total)**	**N (cases)**	**N (total)**	
**Diseases of the nervous system**		**G00 - G99**	**1465**	**99928**	**517**	**22205**	**86**	**2068**	**141**	**4107**	**102**	**2959**	**124404**
	Parkinson disease	G20	32	104226	26	23829	6	2563	5	4574	6	3275	130618
	Multiple sclerosis	G35	17	104153	8	23775	4	2557	4	4564	5	3266	130485
	Epilepsy	G40 - G42	41	104140	18	23799	3	2549	3	4557	2	3263	130488
	Headaches	G43 - G44	142	103934	62	23683	16	2531	25	4522	18	3243	130148
	TIA	G45 - G46	251	103824	55	23732	11	2554	15	4558	10	3266	130110
	Sleep disorders	G47	89	103635	41	23541	13	2499	16	4472	12	3202	129675
**Diseases of the eye**		**H00 - H59**	**5412**	**96229**	**1214**	**21925**	**133**	**2365**	**226**	**4211**	**173**	**3006**	**120519**
**Diseases of the ear**		**H60 - H99**	**351**	**102960**	**83**	**23469**	**21**	**2517**	**23**	**4498**	**19**	**3214**	**128946**
**Diseases of the circulatory system**		**I00 - I99**	**5131**	**91179**	**1194**	**20220**	**166**	**2139**	**273**	**3848**	**188**	**2762**	**113538**
	Hypertension	I10 - I15	167	104045	59	23774	5	2555	14	4566	9	3264	130374
	Ischemic heart diseases	I20 - I25	1859	100205	432	22694	59	2420	92	4326	62	3103	125319
	Angina pectoris	I20	392	103046	94	23407	16	2487	26	4453	17	3189	128940
	Myocardial infarction	I21	741	102913	163	23505	23	2533	37	4519	21	3234	128951
	Pulmonary embolism	I26	375	103751	93	23698	17	2536	27	4532	20	3247	129985
	Arrhythmias	I46 - I49	1276	101971	281	23276	34	2501	60	4457	35	3191	127748
	Heart failure	I50	237	104102	87	23780	17	2559	28	4559	16	3267	130441
	Stroke	I60 - I61, I63 - I64	681	103537	172	23641	14	2539	30	4528	22	3243	129717
	Cerebrovascular diseases	I60 - I69	789	103371	193	23592	16	2533	33	4520	26	3237	129496
	Intracerebral haemorrhage	I61	103	104164	23	23820	0	2564	1	4574	0	3276	130548
	Cerebral infarction	I63	494	103828	130	23734	11	2550	24	4550	17	3257	130112
	Arteriosclerosis	I70	61	104166	9	23807	2	2561	5	4568	2	3272	130534
	Deep vein thrombosis	I80 - I82	282	103674	75	23703	14	2548	18	4545	13	3257	129925

© 2023 Frank P et al. *JAMA Psychiatry.*

eTable 16 continued from previous page

**Disease outcome**													
**Disease category (ICD-10 chapter)**	**Diagnosis or diagnostic group***	**ICD10-codes**	**No depression**		**Mild to moderate**		**Moderately severe to severe**		**Inflammation-related symptom profile**		**Obesity-related symptom profile**		**N (total)**
			**N (cases)**	**N (total)**	**N (cases)**	**N (total)**	**N (cases)**	**N (total)**	**N (cases)**	**N (total)**	**N (cases)**	**N (total)**	
**Diseases of the respiratory system**		**J00 - J99**	**2308**	**99315**	**728**	**22197**	**109**	**2320**	**187**	**4150**	**123**	**2984**	**123832**
	Influenza and pneumonia	J09 - J18	1044	103239	324	23513	54	2516	94	4493	65	3227	129268
	Chronic obstructive bronchitis	J43 - J44, J47	195	104046	93	23740	20	2548	36	4548	22	3254	130334
	Asthma	J45 - J46	105	103916	40	23696	5	2539	13	4538	6	3247	130151
**Diseases of the digestive system**		**K00 - K93**	**9922**	**76514**	**2707**	**16065**	**309**	**1566**	**564**	**2836**	**380**	**2064**	**94145**
	Appendicitis	K35	177	103552	39	23612	8	2544	13	4541	11	3248	129708
	Inflammatory bowel disease	K50 - K52	658	101794	224	22999	29	2425	55	4333	35	3105	127218
	Diseases of liver	K70 - K77	157	103987	59	23754	10	2547	19	4549	12	3257	130288
	Alcoholic liver disease	K70	13	104223	10	23831	1	2564	3	4576	2	3277	130618
	Pancreatitis	K85	103	103967	46	23750	4	2547	11	4545	6	3257	130264
**Diseases of the skin**		**L00 - L99**	**2331**	**97288**	**534**	**22028**	**51**	**2312**	**98**	**4147**	**61**	**2983**	**121628**
	Skin infections and eczema	L00 - L08, L20 - L30	623	102487	191	23279	27	2474	52	4431	27	3174	128240
**Diseases of the musculoskeletal system**		**M00 - M99**	**6354**	**86683**	**1842**	**18972**	**230**	**1875**	**412**	**3389**	**272**	**2459**	**107530**
	Rheumatoid arthritis and related disorders	M05-M06, M08, M13, M30-M35, M45	281	103567	98	23599	15	2510	33	4502	20	3219	129676
	Gout	M10	31	104187	10	23825	4	2563	5	4572	4	3272	130575
	Osteoarthritis	M15 - M19	2937	98532	812	22285	97	2360	169	4215	117	3043	123177
	Sciatica	M50 - M51	341	103050	149	23348	23	2444	46	4401	23	3148	128842
	Back pain	M54	536	102710	253	23216	49	2404	84	4344	56	3126	128330
	Soft tissue disorders	M60 - M79	1760	98260	499	22108	66	2289	117	4098	73	2960	122657

© 2023 Frank P et al. *JAMA Psychiatry.*

eTable 16 continued from previous page

**Disease outcome**													
**Disease category (ICD-10 chapter)**	**Diagnosis or diagnostic group***	**ICD10-codes**	**No depression**		**Mild to moderate**		**Moderately severe to severe**		**Inflammation-related symptom profile**		**Obesity-related symptom profile**		**N (total)**
			**N (cases)**	**N (total)**	**N (cases)**	**N (total)**	**N (cases)**	**N (total)**	**N (cases)**	**N (total)**	**N (cases)**	**N (total)**	
**Diseases of the genitourinary system**		**N00 - N99**	**3722**	**86603**	**1021**	**18657**	**140**	**1847**	**223**	**3358**	**169**	**2425**	**107107**
	Renal failure	N17 - N19	258	104040	94	23763	20	2553	28	4549	19	3266	130356
**Pregnancy complications**		**O00 - O29**	**1**	**53817**	**1**	**13955**	**0**	**1478**	**1**	**2713**	**0**	**1870**	**69250**
	Spontaneous abortion	O03	1	55032	0	14462	0	1549	0	2845	0	1953	71043
	Hypertension in pregnancy	O13 - O16	0	55200	1	14516	0	1550	1	2847	0	1956	71266
	Diabetes in pregnancy	O24	0	55375	0	14587	0	1567	0	2879	0	1974	71529
**Miscellaneous**	Circulatory and respiratory symptoms	R00 - R09	2418	97313	767	21455	110	2186	180	3924	131	2846	120954
	Digestive and abdominal symptoms	R10 - R19	2856	96426	951	21124	130	2100	210	3822	151	2750	119650
	Injury	S00 - T35	2944	96474	794	21772	110	2293	188	4103	136	2924	120539
	Poisoning	T366 - T65	76	103932	37	23633	18	2456	23	4446	16	3166	130021
	Road accidents	V01 - V99	0	104243	0	23843	0	2566	0	4578	0	3278	130652
	Falls	W00 - W19	7	104243	4	23842	2	2566	3	4578	0	3278	130651

© 2023 Frank P et al. *JAMA Psychiatry.*

eAppendix. STROBE Statement: Checklist of items that should be included in reports of observational studies

Section/Topic	Item No	Recommendation	Reported on Page No
**Title and abstract**	1	(*a*) Indicate the study's design with a commonly used term in the title or the abstract	3-4
		(*b*) Provide in the abstract an informative and balanced summary of what was done and what was found	3-4
**Introduction**			
Background/rationale	2	Explain the scientific background and rationale for the investigation being reported	5
Objectives	3	State specific objectives, including any prespecified hypotheses	5-6
**Methods**			
Study design	4	Present key elements of study design early in the paper	6-8
Setting	5	Describe the setting, locations, and relevant dates, including periods of recruitment, exposure, follow-up, and data collection	6
Participants	6	*Cohort study*—Give the eligibility criteria, and the sources and methods of selection of participants. Describe methods of follow-up	6
		(*b*) *Cohort study*—For matched studies, give matching criteria and number of exposed and unexposed<br>*Case-control study*—For matched studies, give matching criteria and the number of controls per case	NA
Variables	7	Clearly define all outcomes, exposures, predictors, potential confounders, and effect modifiers. Give diagnostic criteria, if applicable	7-8
Data sources/measurement	8*	For each variable of interest, give sources of data and details of methods of assessment (measurement). Describe comparability of assessment methods if there is more than one group	7-8
Bias	9	Describe any efforts to address potential sources of bias	9,14,16
Study size	10	Explain how the study size was arrived at	6, Figure 1
Quantitative variables	11	Explain how quantitative variables were handled in the analyses. If applicable, describe which groupings were chosen and why	6-9
Statistical methods	12	(*a*) Describe all statistical methods, including those used to control for confounding	9
		(*b*) Describe any methods used to examine subgroups and interactions	9
		(*c*) Explain how missing data were addressed	9
		(*d*) *Cohort study*—If applicable, explain how loss to follow-up was addressed<br>*Cross-sectional study*—If applicable, describe analytical methods taking account of sampling strategy	8
		(*e*) Describe any sensitivity analyses	9

Section/Topic	Item No	Recommendation	Reported on Page No
**Results**			
Participants	13*	(a) Report numbers of individuals at each stage of study—eg numbers potentially eligible, examined for eligibility, confirmed eligible, included in the study, completing follow-up, and analysed	10
		(b) Give reasons for non-participation at each stage	10
		(c) Consider use of a flow diagram	Figure 1
Descriptive data	14*	(a) Give characteristics of study participants (eg demographic, clinical, social) and information on exposures and potential confounders	10, Table 1, appendix
		(b) Indicate number of participants with missing data for each variable of interest	appendix
		(c) *Cohort study*—Summarise follow-up time (eg, average and total amount)	8
Outcome data	15*	*Cohort study*—Report numbers of outcome events or summary measures over time	10-13, appendix
		Case-control study—Report numbers in each exposure category, or summary measures of exposure	NA
		Cross-sectional study—Report numbers of outcome events or summary measures	10-13, appendix
Main results	16	(*a*) Give unadjusted estimates and, if applicable, confounder-adjusted estimates and their precision (eg, 95% confidence interval). Make clear which confounders were adjusted for and why they were included	10-13, appendix
		(*b*) Report category boundaries when continuous variables were categorized	10-13
		(*c*) If relevant, consider translating estimates of relative risk into absolute risk for a meaningful time period	12
Other analyses	17	Report other analyses done—eg analyses of subgroups and interactions, and sensitivity analyses	10-13
**Discussion**			
Key results	18	Summarise key results with reference to study objectives	13
Limitations	19	Discuss limitations of the study, taking into account sources of potential bias or imprecision. Discuss both direction and magnitude of any potential bias	16-17
Interpretation	20	Give a cautious overall interpretation of results considering objectives, limitations, multiplicity of analyses, results from similar studies, and other relevant evidence	16
Generalisability	21	Discuss the generalisability (external validity) of the study results	16

© 2023 Frank P et al. *JAMA Psychiatry.*

Other Information			
Funding	22	Give the source of funding and the role of the funders for the present study and, if applicable, for the original study on which the present article is based	1

**Give information separately for cases and controls in case-control studies and, if applicable, for exposed and unexposed groups in cohort and cross-sectional studies.*

Note: An Explanation and Elaboration article discusses each checklist item and gives methodological background and published examples of transparent reporting. The STROBE checklist is best used in conjunction with this article (freely available on the Web sites of PLoS Medicine at http://www.plosmedicine.org/, Annals of Internal Medicine at http://www.annals.org/, and Epidemiology at http://www.epidem.com/). Information on the STROBE Initiative is available at www.strobe-statement.org.

© 2023 Frank P et al. *JAMA Psychiatry.*
