# Supplementary material for: Association Between Depression and Physical Conditions Requiring Hospitalization
Source: JAMA Psychiatry. 2023 May 3;80(7):690–9. doi: 10.1001/jamapsychiatry.2023.0777 (PMC10157511; doi:10.1001/jamapsychiatry.2023.0777)
Supplement: Supplement 2. — Data sharing statement [file jamapsychiatry-e230777-s002.pdf]

Data Sharing Statement

Frank. Association Between Depression and Physical Conditions Requiring Hospitalization. *JAMA Psychiatry*. Published May 03, 2023. doi:10.1001/jamapsychiatry.2023.0777

Data
Data available: No

Additional Information
Explanation for why data not available: Syntax for data analysis is provided in the appendix. Our data protection agreements with the participating cohort studies do not allow us to share individual-level data from these studies to third parties. Pre-existing individual-level data access policies for each of the participating cohort studies specify that research data requests can be submitted to each steering committee; these will be promptly reviewed for confidentiality, data protection issues, or intellectual property restrictions and will not unreasonably be refused. Researchers registered with UK Biobank can apply for access to the database by completing an application. This must include a summary of the research plan, data-fields required, any new data or variables that will be generated, and payment to cover the incremental costs of servicing an application (https://www.ukbiobank.ac.uk/enable-your-research/apply-for-access).
